# Occupational Success Across the Lifespan: On the Differential Importance of Childhood Intelligence, Social Background, and Education Across Occupational Development

**DOI:** 10.3390/jintelligence13030032

**Published:** 2025-03-06

**Authors:** Georg Karl Deutschmann, Michael Becker, Yi-Jhen Wu

**Affiliations:** 1 Center for Research on Education and School Development, TU Dortmund University, 44227 Dortmund, Germany; michael.becker@tu-dortmund.de (M.B.);; 2 DIPF | Leibniz Institute for Research and Information in Education, 60323 Frankfurt, Germany

**Keywords:** late childhood intelligence, socioeconomic background, quantity and quality of education, education as a mediator, socioeconomic success, lifespan perspective, longitudinal study

## Abstract

What shapes (occupational) success in later life? This study examines the differential importance of intelligence in late childhood, socioeconomic background, and education across later occupations. The quantity and quality of educational success are thought to mediate the other dimensions. We analyzed data from *N* = 4387 participants in a German longitudinal large-scale study in multiple regression and mediation models to examine how childhood intelligence and socioeconomic background predict income and occupational status at different career stages. Both childhood intelligence and socioeconomic background predict status and income in adulthood, with childhood intelligence being the stronger predictor. However, education is an even stronger predictor and—once included in the model—mediates virtually all effects of childhood intelligence and socioeconomic background. This pattern remains stable across career stages, and education has unique effects on income and occupational status in later work life, even when controlling for work experience. Our results emphasize the pivotal role of education in transitioning to the labor market and further development at work, even at later career stages. Given the stronger link between childhood intelligence and educational success in Germany than in other countries, we find that Germany is one of the more intelligence-driven systems.

## 1. Introduction

Modern society is based on the meritocratic promise that performance leads to success. To assess whether this societal promise is kept or broken, scholars need to examine occupational success and how it is gained. What factors predict later occupational development and lead to higher income and occupational status? Research on the prediction of income and occupational status has been fragmentary and unsystematic (Hasl et al. 2019) in various ways. Indeed, there is disagreement on which factors are the main drivers of later income and occupational status. Many approaches have focused on childhood intelligence (Anger and Heineck 2006; Sorjonen et al. 2012) and socioeconomic background (Schoon 2008) or a combination of both (Hegelund et al. 2020; Settersten and Gannon 2005), as they seem to be the most important drivers of occupational success. Studies in various countries have found different effect sizes when relating predictors to later outcomes (Sorjonen et al. 2012; Zax and Rees 2002). Recent results have also highlighted the crucial importance of education for later success. These results indicate that education partially mediates between childhood intelligence, socioeconomic background, and later income and occupational status (Becker et al. 2019; Becker and Tetzner 2021). Nevertheless, few studies have considered how childhood intelligence, socioeconomic background, and education predict income and occupational status at the same time.

It is furthermore unclear whether childhood intelligence, socioeconomic background, and education predict income and occupational status homogeneously over time or to what extent the prediction differs across the lifespan. Ganzach (2011) and Strenze (2007) identified factors that could have differential influence at different stages in life. Later career development could be driven by cognitive abilities, while the socioeconomic background may offer resources that are especially useful at the beginning of the career. However, many studies have focused on just two time points, such as school-related predictors of middle-adulthood outcomes (Damian et al. 2016; Johnson et al. 1983; Lubinski et al. 2014; Schoon 2010; Spengler et al. 2015), partially disregarding development across the lifespan (see also Hasl et al. 2022; Settersten and Gannon 2005) or ignoring the important transition to adulthood and entering the labor market (Bleidorn 2012; Buchmann and Kriesi 2011; Hanushek et al. 2017).

We aim to clarify the extent to which childhood intelligence, socioeconomic background, and education predict income and occupational status and how the prediction varies over time. We modeled (to our knowledge for the first time) the longitudinal effects of socioeconomic background, cognitive ability, and education on two dimensions of later occupational success at multiple time points in adulthood. Using a hierarchical step-by-step procedure, we show the impact the inclusion of each factor has on the prediction of later occupational success. We used school-related variables to predict income and occupational status as dimensions of occupational success at three-time points: at entry into the job market and then twice during adulthood (at the age of about 30 and 40 years). We used data from Germany—which is exemplary for countries with specialized educational certificates (Bol and van de Werfhorst 2013), and less researched than the US and the UK.

### 1.1. Predicting Occupational Success

Income and the prestige associated with occupational status are two paramount indicators determining occupational success during adulthood (Fujishiro et al. 2010). To understand how occupational success is attained and how it changes across the lifespan, psychological and sociological research has used integrative theoretical models, particularly human capital theory (Mincer 1958; Becker 2009), the Wisconsin model (Crouse et al. 1979; Sewell et al. 1969), and theories developed from them (e.g., Credé and Kuncel 2008; Pfeffer 2011).

Human capital theory assumes that investments of resources are associated with occupational (and thus economic) success (Sweetland 1996). These resources mainly stem from an individual’s cognitive abilities, socioeconomic background, and education (Fitzsimons 2017). The relationship of invested resources to later success is straightforward: the more resources invested, the more human capital is accumulated. As a result, individuals have a high likelihood of success in the labor market. The Wisconsin model also identifies cognitive abilities and socioeconomic background as core factors for later occupational success but underscores the role of education in cognitive abilities and SES (Warren et al. 2002). While cognitive abilities and socioeconomic background are assumed to have a direct effect on occupational success (Bernardi et al. 2019; Conley and Gifford 2006), researchers see education as a key link between cognitive abilities and socioeconomic background in youth and success in later adulthood (Belley and Lochner 2007; Bozick et al. 2010; Morgan and Kim 2006). Cognitive abilities and socioeconomic background have an impact on educational attainment, and educational attainment has an impact on occupational performance and success.

Contemporary research on longitudinal development focuses on these three core factors that may influence later success. The first of these is an individual’s cognitive abilities, which is a factor in both human capital theory and the Wisconsin model. The link between cognitive abilities and later occupational success has received a lot of attention (Brandt et al. 2021; Brown et al. 2021; Gottfredson 2003; Schmidt and Hunter 2004). Higher cognitive abilities lead to better performance in assessments and job interviews and a higher starting salary and job position (Anger and Heineck 2006; Bergman et al. 2014; Murtza et al. 2021; Shavit and Müller 2003). Furthermore, cognitive abilities and job productivity are closely related (Murtza et al. 2021; Wai 2014); the higher the job productivity, the higher the wages and the occupational position (Byington and Felps 2010). Therefore, productivity, accumulated cognitive abilities, and the capability to use those skills at work are connected to occupational success (Cunha and Heckman 2007). Cognitive abilities in adult life are significantly influenced by childhood intelligence. While intelligence seems to remain stable across adulthood, it is highly predictive of later occupational performance (Becker et al. 2019; Bottenhorn et al. 2021; Lubinski et al. 2014). A meta-analysis showed that childhood intelligence is a very strong predictor of later occupational success (e.g., size of correlations/effect size), with significant correlations on both later occupational status and income (Strenze 2007).

Socioeconomic background is regarded as a source of human capital and—as stated in the Wisconsin model—a key factor in occupational status. The socioeconomic background of an individual’s family can support or hinder them in attaining highly paid jobs and prestigious occupational positions. Socioeconomically and socioculturally affluent parents are likely to place their children in promising school environments and give them opportunities in adult life (Cunha and Heckman 2007; Hegelund et al. 2020; Stumm and Plomin 2015). Even without direct action, affluent socioeconomic environments can offer valuable prospects for further cognitive and non-cognitive development (e.g., parents can purchase private lessons or supplemental coaching for underperforming children; Davis-Kean 2005). While different studies have found a varying influence of socioeconomic background on later occupational success (Deary et al. 2005; Judge et al. 2010; Spengler et al. 2018; Zax and Rees 2002), they all consistently show a significant positive relationship between socioeconomic background and later occupational success. The variation in effect sizes may partly result from differential modeling of socioeconomic background—not all studies use latent models of socioeconomic background (e.g., Zax and Rees 2002). Nevertheless, Strenze’s (2007) meta-analysis shows that socioeconomic background was significantly correlated with both later income and occupational status.

The third major predictor of occupational success, found in both human capital theory and the Wisconsin model, is education. The Wisconsin model claims that high cognitive abilities lead to better educational performance and educational success. Research also indicates that the more capital is available, the more can be invested in education (and indirectly to educational success) (Fitzsimons 2017; Maaz et al. 2014; Schulz et al. 2017). In this way, cognitive abilities and socioeconomic background interact with educational attainment and later educational outcomes (Deary et al. 2007; Georg 2009; Schneider and Preckel 2017). Many studies view educational outcomes as indicators of socioeconomic success (Strenze 2007), but past educational outcomes also influence both occupational prestige and income (Ganzach 2011). There are various paths that seem relevant. Education is an ongoing process of gaining human capital in terms of cognitive, psychological, social, and economic resources (e.g., by gaining useful skills, abilities, and knowledge or even establishing professional networks). The more human capital an individual can deploy, the greater the reward in terms of (occupational) success (Becker 2009; Heckman and Carneiro 2003). Young people also acquire certificates on their path through the educational system. These educational certificates function as a signal for potential employers, directly affecting later occupational position and income (“signal theory”, see: Arrow 1973; Spence 1973; Konietzka and Hensel 2017).

Upon entering the job market, applicants have no record of productivity from previous work experience. When recruiting, the potential employer estimates expected job productivity by screening the applicants’ educational certificates (Altonji and Pierret 2001). While there is an ongoing discourse about the possibilities of empirically distinguishing between the effects owing to signaling and human capital, there is a great deal of empirical evidence to show that both effects exist and influence later occupational success (Huntington-Klein 2021). On a systemic level, relying on certificates as signals may lead to social closure (Bills 2003), meaning educational certificates can limit access to highly paid and prestigious occupational positions. Educational attainment and success are thus seen as a social sorting mechanism—a theory known as the filter theory (Arrow 1973). From a merit point of view, strict filtering can be problematic. In spite of actual cognitive and non-cognitive abilities, a lack of appropriate certificates impedes potentially highly productive individuals from reaching prestigious positions.

All three pathways suggest that differences in education lead to differences in income and occupational status in later life and offer valuable insights into the underlying mechanisms (Collins et al. 2019). Recent empirical findings confirm education is a mediator between cognitive abilities, socioeconomic background, and later income and occupational prestige (Becker et al. 2019; Bukodi et al. 2014; Marks 2015; Schoon 2008; Spengler et al. 2015).

### 1.2. Entering the Labor Market and Later Development in Income and Occupational Status Across Working Life

A lifespan is a series of events, conditions, and roles that are causally connected (Blossfeld 1989; DiPrete and Eirich 2006; Giele and Elder 1998; Mayer 2009; Wingens 2022). According to lifespan theory, each individual constantly develops self-agency and becomes more independent from parental influence over time by establishing their own social network (Gecas 2003). This implies that occupational success may be influenced by differential factors at different stages of the lifespan and that the influence of childhood intelligence, socioeconomic background, and education may vary across these stages. From this lifespan perspective, we can identify two major differential stages of work life in early and middle adulthood. Scholars have described them as the most important processes for gaining and developing occupational success (Blossfeld 1989): the transition from an educational to an occupational context upon entry into the labor market (Buchmann and Kriesi 2011; Kerckhoff 2003) and the period following an individual finding their position in the labor market (Baltes 1987; Heinz 2003; Mortimer et al. 2003). Both processes are interrelated.

Entry into the labor market is a central transition in the individual life course (Bleidorn 2012; Buchmann and Steinhoff 2017; Hanushek et al. 2017). Childhood intelligence and socioeconomic background influence the transition into the labor market. While cognitive abilities assist performance in assessments and job interviews (Geißler 2018), parental networks and personal connections can often surpass conventional application paths (Banaszczuk 2017; Jacob and Klein 2013). Signaling theory demonstrates how educational certificates also play a major role in the transition, as they serve as indicators of an individual’s prospective productivity. Childhood intelligence and socioeconomic background both influence educational success and certificates (Bottenhorn et al. 2021; Maaz et al. 2014; Schneider and Preckel 2017; Stumm and Plomin 2015). Recent findings have shown strong effects of education on occupational prestige and income at entry into the labor market (Forster et al. 2016; Hanushek et al. 2017; Liwiński and Pastore 2021; Schiener 2010). However, the extent to which childhood intelligence, socioeconomic background, and education each contribute to the transition to the labor market is still under-researched.

Earlier career stages often determine how an individual’s working life will develop (Blossfeld 1989), and income and occupational status usually increase in the course of a career. Previous occupational status and income influence later status and income (Dannefer 2003; Hasl et al. 2022; Ludwig 1996). While only a few studies have examined different periods of occupational success over time, there are some indicators that the change is not homogenous; it varies across different stages in a career. Recent studies have discussed three different approaches that could explain why change over time is not homogenous.

First, after labor market entry, the employer does not need to estimate productivity but can evaluate the employee’s productivity directly and adjust the salary and job position accordingly. Since cognitive abilities and productivity are interrelated, strong childhood intelligence predicts later occupational success (Anger and Heineck 2006; Cawley et al. 2001; Kramer 2009; Lin et al. 2016). Studies have shown that the influence of cognitive abilities and socioeconomic background on occupational success varies across the individual life course (Baltes 1987; Georg 2009; Settersten and Gannon 2005). Ganzach (2011) found a major influence of socioeconomic background on occupational status and income at entry into the labor market. Strenze (2007) and Ganzach (2011) found that cognitive abilities have a growing influence on further progression at work, while the influence of socioeconomic background declines across the lifespan.

Second, previous occupational status and income affect later occupational status and income. Meritocratic theory states that occupational success emerges from productivity (Weiss 1995), while productivity itself is seen as a realization of human capital (Becker 2009). If we combine human capital and merit theory, we would expect productivity to be reciprocally related to occupational success. A steady source of human capital is derived from the increasing experience and specialized work knowledge gained over time in a position. This accumulated human capital leads to higher work productivity (Franz 2013). In this way, income and occupational status may advance over time on an autoregressive path; experience gained at work leads to higher productivity, and higher productivity leads to higher income and occupational status. Studies have confirmed that the change in income and occupational status aligns with productivity at work (Altonji and Pierret 2001), but few studies have controlled for work experience itself.

Third, education affects occupational status and income at later stages in a career. Competencies acquired by education are sources of human capital and also have a positive effect on job performance and occupational success (Cunha and Heckman 2007; Heckman and Carneiro 2003). Educational performance and the resulting educational success are influenced by childhood intelligence and socioeconomic background (Bottenhorn et al. 2021; Deary et al. 2007; Schneider and Preckel 2017; the hypothesis of the Wisconsin model). In this way, educational success, which is partly influenced by both childhood intelligence and socioeconomic background, is one of the most important predictors of income and occupational status, both at the start and later in a career. Therefore, education might mediate the relationships between childhood intelligence, socioeconomic background, and later occupational status. Becker et al. (2019) found that education overtakes predictive power from childhood intelligence and socioeconomic background and predicts income and occupational status even more prominently than childhood intelligence or socioeconomic background as such. However, it is not known whether the influence of education on income and occupational status remains steady over time. As work experience gains importance over time, the influence of education on income and occupational status could gradually weaken.

### 1.3. Different National Contexts

Education systems and educational certificates vary between countries. Some countries have a broad and highly structured vocational education sector (DiPrete et al. 2017); high vocational training leads to highly specific certificates (Hanushek et al. 2017). Referring to signal theory, the more specific the certificate, the easier it is to estimate an individual’s prospective productivity in the specific job position, making the process of matching potential employers and employees smoother (Gangl 2003; Konietzka and Hensel 2017). Therefore, it is easier to transition into the labor market in countries with highly specific certificates (Becker et al. 2019; Sorjonen et al. 2012) than in countries with less specific certificates (Shavit and Müller 2003). Specific certificates can more clearly predict later performance in specific work tasks, making education a powerful predictor of change at work in countries with specific certificates. Studies have found a major effect of education and no effect of socioeconomic background in countries such as Germany and Sweden (Bukodi et al. 2014; Bukodi et al. 2017).

In countries with less specific certificates, potential employers often use multiple interviews and assessments to estimate the individual’s prospective productivity (Rothwell and Lindholm 1999; Rothwell et al. 2025). Performance in those interviews and tests is more important in the process of acquiring a job than just educational certificates (Heimann et al. 2021). Therefore, education is less predictive in those countries with less specific certificates as they give no clear criteria for sorting people. Cognitive abilities or socioeconomic background might drive actual work performance and predominate in determining a career path. Studies in the UK and USA have shown a strong effect of childhood intelligence and socioeconomic background on income and occupational status in later life (Rumberger 2010; Spengler et al. 2018; Zax and Rees 2002). We can thus hypothesize that education will be more predictive in countries with highly specialized certificates than in countries with less specialized certificates. However, most research still stems from systems with low structuring (Bol and van de Werfhorst 2013). We still do not know if education is a more powerful driver of occupational success than cognitive abilities and socioeconomic background in countries with highly specialized certificates.

### 1.4. The Study

Previous research has shown that childhood intelligence, socioeconomic background, and education influence later income and occupational status. However, research has been unsystematic and partially contradictory. First, it is unclear to what extent childhood intelligence, socioeconomic background, and education predict income and occupational status at different stages in a career (Question 1). Second, it is unclear how the predictive power of childhood intelligence, socioeconomic background, and education changes over the different stages of a career (Question 2). Third, it is unclear to what extent preceding income and occupational status fully predict later income and occupational status or if there are any unique effects of childhood intelligence, socioeconomic background, and education on income and occupational status in later work life beyond the preceding income and occupational status (Question 3).

To answer these questions, we used data from the longitudinal study “Educational Careers and Psychosocial Development in Adolescence and Young Adulthood” (Bildungsverläufe und psychosoziale Entwicklung im Jugendalter und jungen Erwachsenenalter or BIJU, Baumert et al. 1996). The BIJU study followed its participants from youth to the age of nearly 40. These data provide insights into important stages in the progression from late childhood to young adulthood and into adulthood itself. Our study proceeds from the latest international research on individual progression from late childhood to adulthood and expands it by systematically focusing on Germany. We examined income and occupational status across work life at three time points, with the first time point at the entry to the labor market, the second at the age of 30, and the third at the age of nearly 40.

For each time point, we ran predictions on occupational status and income based on late childhood intelligence, socioeconomic background, and education (Question 1). According to human capital theory and the psychological view on individual differences (Brandt et al. 2021; Brown et al. 2021; Judge et al. 2010; Spengler et al. 2018), we expected that income, occupational status, and educational success would be related to intelligence and socioeconomic background (**Hypothesis 1.1**). Considering recent findings, we expected education to mediate between late childhood intelligence, socioeconomic background, and later income and occupational status (Becker et al. 2019; Hasl et al. 2022; Karlson and Birkelund 2019). In a direct comparison between models that include education as a mediator and models that omit it, we expected that education would explain some effects of socioeconomic background and childhood intelligence (**Hypothesis 1.2**) and would be an even stronger predictor of later income and occupational status than childhood intelligence and socioeconomic background (**Hypothesis 1.3**).

Focusing on changes across time points (Question 2), we addressed the changing effects of childhood intelligence and socioeconomic background on later income and occupational status (**Hypothesis 2.1**) as an open question. Following the signal/filter theory, we expected to find strong effects of education on both income and occupational prestige at entry into the labor market but also assumed no change or even a decline in the influence of education across the lifespan (**Hypothesis 2.2**).

Addressing the third question of whether earlier income and status can completely explain later income and occupational status (Question 3), we examined the unique effects of cognitive abilities, socioeconomic background, and education after entry into the labor market. Human capital theory suggests that all three predictors are factors for human capital that lead to higher productivity and, therefore, higher income and occupational status. Accordingly, we assumed that even after modeling preceding income and occupational status, significant unique effects of socioeconomic background, childhood intelligence, and education would remain at each time point (**Hypothesis 3.1**).

## 2. Materials and Method

### 2.1. Sample

We used the dataset of the large-scale study “Educational Trajectories and Psychosocial Development in Adolescence and Young Adulthood” (BIJU; Baumert et al. 1996). As part of this study, data were collected in four German federal states: Berlin, Mecklenburg-Western Pomerania, North Rhine–Westphalia, and Saxony–Anhalt. Data collection took place from 1991 (Wave 1: school years/12 years of age) to 2018 (Wave 8: working life/approx. 40 years of age). We used data from Waves 7 and 8 to model three time points in working life. Time point 1 contains retrospective information on career entry, and time points 2 and 3 contain current information on working life at the ages of 30 and 40. We also used information on childhood intelligence from Waves 1 and 2 and information on socioeconomic background from Wave 4. As information on education was updated throughout the waves, we used the latest available information for each individual.

At the start (Waves 1 and 2), the BIJU study comprised a sample of *N* = 8043. After longitudinal dropouts, e.g., due to students changing schools or completing their school career (Wave 4: *N* = 5386), the number of participants was increased to *N* = 8061 by oversampling for the 5th wave. In Wave 7, at the age of just under 30 years, information is available from *N* = 4130, and in Wave 8, at the age of just under 40 years, from *N* = 2687 participants. Panel mortality and sample selectivity are comparable with other longitudinal large-scale studies (Spengler et al. 2018).^1^ Our analytical sample from the longitudinal section of the BIJU study includes all individuals for whom addresses were available for Wave 8 and who had provided information on income and occupational status for at least one point in time. For the final sample, we used data from *N* = 4374 people, 37.1% of whom were male. Further descriptive data are listed in Table 1. 

The BIJU study was conducted in accordance with the APA guidelines for social research. Participation in this study was voluntary. Written consent was obtained from all subjects and their parents. The information from the first waves (1–4) was collected at schools. Data from wave 5 were collected directly at schools or by post, depending on the level of educational participation. After the school period (Wave 6 and subsequent waves), the survey was conducted by post. This study and all materials and procedures used were approved by the ethics committee of the Max Planck Institute for Human Development and the education ministries of the respective federal states (Baumert et al. 1996).

### 2.2. Instruments

#### 2.2.1. Income and Occupational Status as Indicators of Occupational Success

Information on income and occupational prestige was taken from Wave 7 (2010) and Wave 8 (2018). As well as information on actual occupational status and income, both waves offer retrospective information on labor market entry. All data were self-reported by the participants in the surveys.

##### Income and Occupational Status

We used information on monthly pre-tax income in euros (€). To avoid bias based on extreme values and the distributional shape, we used a logarithmic transformation of income. This is in line with related studies (Becker et al. 2019; Damian et al. 2016; Strenze 2007).

Occupational status was assessed by free text items and then coded according to the 2008 version of the International Standard Classification of Occupations (ISCO 2008-Index; International Labour Office 2012). To obtain a metric score for occupational status, the ISCO codes were then transformed according to the International Socio-Economic Index of Occupational Status (ISEI-Index; Ganzeboom and Treiman 2003; Ganzeboom 2010). The index ranges from 14.21 (=cleaner) to 88.96 (=judge), with 56.66 as the mean value at job entry. The *Data Processing Center* (DPC, Hamburg) carried out ISCO coding and ISEI transformation.

To give a practically relevant idea of the metric of both measures, a value of 7.34 at job entry in logarithmized monthly pre-tax income corresponds to 1540 euros per month pre-tax income (see Table 1). An ISEI-coded occupational status with values near 57 at job entry represents a variety of occupations: nurse, general manager in a small business, commercial agent, or legal clerk (see Ganzeboom and Treiman 2003)^2^.

#### 2.2.2. Baseline Predictors

##### Socio-Economic Background

We modeled socioeconomic background from information on parents’ occupational status and level of education. Information on occupational status was openly requested from participants in Waves 1–5 and coded according to ISCO 88 (International Labour Organization 1990). In our analyses, we used values recoded according to the ISEI index. ISCO coding and transformation into ISEI values were carried out by the Center for Surveys, Methods, and Analyses (*Zentrum für Umfragen, Methoden und Analysen* or ZUMA, Mannheim). Detailed additional codes for occupation coding in Germany were considered during coding (Geis and Körber 2011). Information on parents’ level of education included the number of diplomas and higher education qualifications in the parental household. The information from the mother and father was combined so that two pieces of information on education in the parental household were taken into account, each ranging from 0 (neither parent has a high school diploma/university degree) to 2 (both parents have a high school diploma/university degree). All indicators were used as indicators of a latent socioeconomic background factor, meaning that the criteria of good model fit were met (see Section 2.3, “Statistical Analyses”).

##### Childhood Intelligence

We operationalized late childhood intelligence via two subscales of the Cognitive Capacity Test (*Kognitiver Fähigkeitentest*, or KFT 4–13+; Heller et al. 1985) and two subtests of the Intelligence Structure Test (IST; Amthauer 1955) from multiple time points in school and modeled a latent factor. Both tests continue to be used in updated versions in Germany (Amthauer et al. 2001; Heller and Perleth 2000; Liepmann et al. 2007) but have been updated/reformed over the years since they were used in the early waves of BIJU. Since no substantial adjustment was applied to the subtests we use, the recent updates to the tests have no impact on our study. The reliability varied between subscales but not between time points. The figural KFT resulted in Cronbach’s α = 0.91, and the verbal KFT a Cronbach’s α = 0.82. The A and B versions of the IST spatial subscale resulted in Cronbach’s α = 0.68 and 0.74, respectively. Cronbach’s α for the numeric subscale of the IST was 0.90. The four measurements were used as indicators of a latent intelligence factor, meaning that the criteria of good model fit were met (see Section 2.3 Statistical Analyses).

#### 2.2.3. Education as a Mediator

We used information on general and vocational education. The combined quantity of general and vocational education was coded in weighted educational years, according to the Comparative Analysis of Social Mobility in Industrial Nations index (CASMIN; König et al. 1988). Information on education was gathered in every wave and updated throughout this study. We used the most recent information for each individual. The average number of educational years in our sample is *M* = 16.25 (*SD* = 2.81). We also used information on the quality of educational success, GPAs, and the highest vocational diploma of each individual.

#### 2.2.4. Control Variables

Development in occupational status and income is multidimensionally influenced by gender, immigration background, and local system-specific effects (Cheng 2014; Diehl et al. 2016). We considered all three aspects. We controlled for immigration background and local system-specific effects as dummy variables in all models. We controlled for data origin from the federal states (0 = federal states of the former West Germany, 1 = “new” East German federal states). To control for immigration background, we distinguished between both parents having been born in Germany or at least one parent having been born abroad (0 = no immigration background, 1 = immigration background). We also modeled gender dichotomously (male, female) and calculated the models separately in the subgroups (Table A1 in Appendix A).

### 2.3. Statistical Analyses

**Figure 1:**
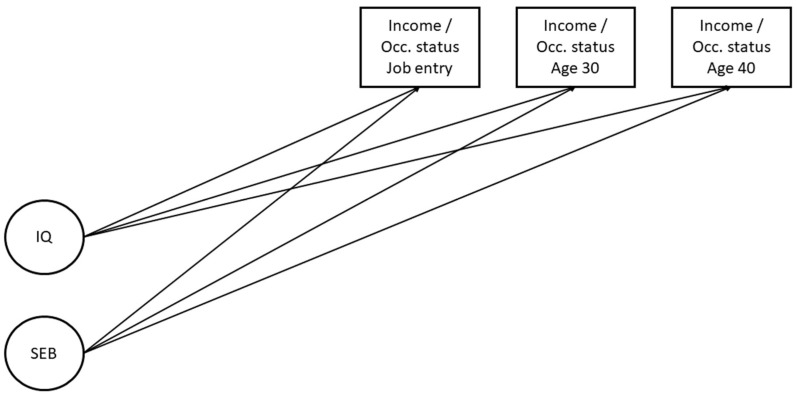
Regression of income and occupational status on childhood intelligence and socioeconomic background. IQ = late childhood intelligence. SEB = socioeconomic background. Income = monthly gross income, logarithmized. Occ. status = occupational status, coded according to the ISEI Index.

**Figure 2:**
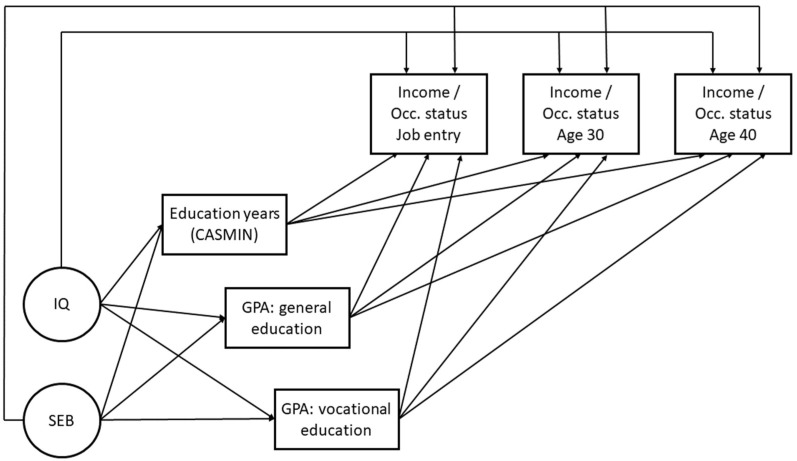
Regression of income and occupational status on childhood intelligence and socioeconomic background, mediated by education. IQ = late childhood intelligence. SEB = socioeconomic background. Education years (CASMIN) = general and vocational education years, weighted according to CASMIN. GPA: general education = grade point average of highest certificate in general education. GPA: vocational education = grade point average of highest certificate in vocational education. Income = monthly gross income, logarithmized. Occ. status = occupational status, coded according to ISEI-Index.

First, we addressed the extent to which childhood intelligence, socioeconomic background, and education predict income and occupational status at all three stages in work life (Question 1), using correlations and regressions to examine the effects of the predictors. The first time point is the individual’s entry into the labor market, and the second and third time points are at age 30 and nearly 40. We regressed income and occupational status directly on childhood intelligence and socioeconomic background at each time point separately. Education was modeled as a mediator; income and occupational status are predicted by education, while education itself is regressed on childhood intelligence and socioeconomic background. In our regression models, we focused on the time points when each predictor shows significant effects on income and occupational status (**Hypothesis 1.1**). We contrasted models that only include late childhood intelligence (which we abbreviate below as *IQ*) and socioeconomic background (which we abbreviate below as *SEB*) (see Figure 1) with models that also include education quantity and quality as a mediator (see Figure 2) to determine the impact of education above the smaller model (**Hypotheses 1.2** and **1.3**). Figure 1 shows the conceptual models in which we estimated the predictions of income and occupational success at job entry, age 30, and age 40 by childhood intelligence and socioeconomic background.

Figure 2 shows the conceptual models in which we estimated the prediction of income and occupational status at job entry, age 30 and age 40, by childhood intelligence and socioeconomic background, mediated by quantity and quality of education. The models in Figure 1 and Figure 2 differ with regard to the inclusion of the mediating effects: Comparing the effects in both models shows the mediating effect of quantity and quality of education. If we found a significant effect from childhood intelligence on income at age 30 in the Figure 1 models but the same effect had no significance in the Figure 2 models, we assumed that the (direct) effects found in the Figure 1 model had been explained by education in the Figure 2 model. We interpret this as a mediation of effects between childhood intelligence and income by education.

Second, we investigated how the predictive power of childhood intelligence, socioeconomic background, and education changed over time (Question 2). We determined which predictor showed the strongest effect on income and occupational status at each time point (**Hypothesis 2.1**). We examined whether there were statistically significant changes to the effects across the measured time points by using the chi-square difference test (ꭓ^2^ test). A statistically significant change in effects between two time points indicated an increase or decrease in the predictor’s influence (**Hypothesis 2.2**).

**Figure 3:**
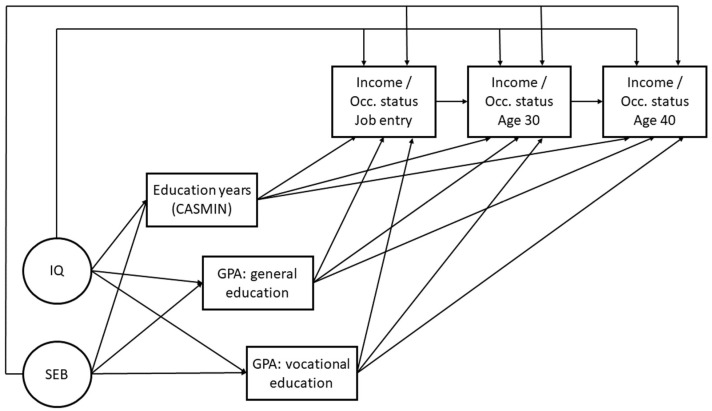
Regression of income and occupational status on childhood intelligence and socioeconomic background, mediated by education, autoregressive paths. IQ = late childhood intelligence. SEB = socioeconomic background. Education years (CASMIN) = general and vocational education years, weighted according to CASMIN. GPA: general education = grade point average of highest certificate in general education. GPA: vocational education = grade point average of highest certificate in vocational education. Income = monthly gross income, logarithmized. Occ. status = occupational status, coded according to ISEI-Index.

Third, we tested if former income and occupational status predict later income and occupational status (Question 3). We established path models of income and occupational status across the three time points, testing the income and occupational status stability and adding socioeconomic background, cognitive abilities, and education at the latter two time points. We regarded statistically significant effects of the predictors as unique individual effects that directly affected the later changes in income and occupational status over and above the effect of experience and performance at work (**Hypothesis 3.1**). Figure 3 shows the conceptual model used for the path models. The direct paths between income and occupational status at job entry and age 30 and between age 30 and age 40 display effects of an autoregressive development of income and occupational status across work life. We assumed significant effects on the other (direct) paths of childhood intelligence, socioeconomic background, and (mediated by) quantity/quality of education to be unique effects on later income and occupational status (cf. **Hypothesis 3.1**).

We modeled childhood intelligence and socioeconomic background as latent measures. Both latent models fulfilled the criteria of good model fit (i.e., SRMR < 0.05, RMSEA < 0.05, and CFI > 0.90; cf. Byrne 2012), with standardized root mean square residual (SRMR) = 0.02, root mean square error of approximation (RMSEA) = 0.03, comparative fit index (CFI) = 0.99 for the latent model of childhood intelligence, and SRMR = 0.02, and with RMSEA = 0.03 and CFI = 0.98 for latent socioeconomic factor.

We also checked whether income and occupational status could be modeled as a combined latent outcome of occupational success. The test for measurement invariance over time showed insufficient model fit on configural invariance. Therefore, we did not model a combined latent measurement of occupational success and treated income and occupational status separately as manifest outcomes.

We used Mplus version 8.6 (Muthén and Muthén 2017) to estimate all measurement, regression, and path models (Full Mplus syntax is provided in the supplementary materials information). All reported models met the criteria for good model fit (Byrne 2012). We used data weights that accounted for the differential sampling probability of the stratified sample (by federal state, type of school, and classes). Robust standard errors accounted for the clustered sampling. Missing values were estimated via the full information maximum likelihood procedure (FIML; see Graham 2009). All results are fully standardized.

## 3. Results

### 3.1. Predicting Income and Occupational Status at Different Stages in Work Life

Regarding the extent to which childhood intelligence, socioeconomic background, and education predict income and occupational status at different stages in work life (Question 1), we first present the absolute effects of each predictor on each outcome (Table 2). Quantity of education (in years) showed a strong statistically significant correlation to cognitive abilities (0.62) and socioeconomic background (0.43). We found statistically significant correlations between childhood intelligence, socioeconomic background, and quantity (years) and quality (GPA) of education with both income and occupational status at every time point. Nevertheless, at each time point, the correlations of quantity of education with income and occupational status were even stronger than the correlations of childhood intelligence and socioeconomic background with income and occupational status (confirming **Hypothesis 1.1**).

To further explore the conjoint relations, we used multivariate regression models to predict income and occupational status by childhood intelligence and socioeconomic background. Table 3 shows the results of these multivariate regressions (cf. Figure 1), with β representing the standardized effect of the predictors (left column: Child. intel. and Soc. background) on the outcomes at the respective time points (left column: Job entry, Age 30 and Age 40). For income, childhood intelligence and socioeconomic background had a statistically significant effect at job entry and age 30. Childhood intelligence (entry: β = 0.21, age 30: β = 0.21) was a more powerful predictor than socioeconomic background (entry: β = 0.12, age 30: β = 0.09). However, at age 40, only socioeconomic background had a statistically significant effect on income (age 40: β = 0.17). For occupational status (Table 3, Occ. Status), statistically significant effects of both predictors were evident at all time points; cognitive abilities (entry: β = 0.42, age 30: β = 0.38, age 40: β = 0.41) were more predictive than socioeconomic background (entry: β = 0.25, age 30: β = 0.27, age 40: β = 0.22).

While most correlations were significant, the standardized effects were between small and medium (Brydges 2019). Even small (differences in) effects can have a large impact on an individual’s life—we used a logarithmized transformation of income and ISEI-coded information on occupational status. The effect of β = 0.21 of childhood intelligence on income at job entry was a difference of 13% per SD in childhood intelligence. On the basis of 1540 euros mean pre-tax monthly income at job entry (logarithmized value 7.34, see Table 1), this meant an additional 200 euros per SD for childhood intelligence. The effect of β = 0.42 for childhood intelligence on occupational status represented a difference of 8 points on the ISEI-Index per SD of childhood intelligence. Taking the example of the mean value of occupational status of 57 (legal clerk or commercial agent), an additional 8 points would lead to a value of 64 (detective inspector or municipal consultant).

Next, we explored the extent to which these results changed once quantity and quality of education were added to the regression models (**Hypotheses 1.2** and **1.3**). Table 4 informs about the multivariate regressions of income and occupational status on childhood intelligence, socioeconomic background, and education as a mediator (cf. Figure 2). All cells, except for the rows for *R*^2^, contain the standardized effects (β) of the predictors (listed in the left column) on the outcomes (listed in the top row) at the different time points (left column). As shown in Table 4, we found significant effects of late childhood intelligence on quantity and quality of education (Education in years: β = 0.54, GPA general education: β = 0.38, GPA vocational education: β = 0.41), while socioeconomic background only had significant effects on the quantity of education (Education in years: β = 0.24). For both income and occupational status, the effects of quantity of education were statistically significant at all time points (Table 4). The effects of socioeconomic background and cognitive abilities were smaller (which means they were partially mediated by education) or not significant (which means they were fully mediated) once quantity and quality of education were included in the prediction, confirming **Hypothesis 1.2**. Education mediated all the effects of socioeconomic background and late childhood intelligence on income. Furthermore, quantity of education was more predictive for occupational status (entry: β = 0.39, age 30: β = 0.50, age 40: β = 0.55) than income (entry: β = 0.24, age 30: β = 0.28, age 40: β = 0.32). Compared with socioeconomic background and late childhood intelligence, education was the most powerful predictor for occupational status and income at all time points, confirming **Hypothesis 1.3**.

### 3.2. Changes in the Predictions over Time

We addressed the second question as to whether the predictive power of late childhood intelligence, socioeconomic background, and quantity and quality of education on income and occupational status changed over time (Question 2) by testing the change in effects. Results from our regression analyses (Table 4) suggested that the predictive power of cognitive abilities, socioeconomic background, and education varied across different time points.

Addressing the topic of whether there was a change in the effects of late childhood intelligence and socioeconomic background on later occupational success (**Hypothesis 2.1**) and whether there was no change or even a decline in the effects of education on occupational success over time (**Hypothesis 2.2**), we used chi-square difference tests (ꭓ^2^ test) to evaluate whether the effects of late childhood intelligence, socioeconomic background and education changed over time (by freely estimating the parameters) or remained stable across time (by constraining the parameters to be equal across time). Table 5 shows the results of the ꭓ^2^ tests for changes in the effects of intelligence, socioeconomic background, and quantity and quality of education for predicting income and occupational status across time in the mediation models shown in Table 4. If the *p*-value was > 05 we assumed that the models with β constrained to be equal across the time points significantly differed from the reference models where β was estimated freely. Based on a significant ꭓ^2^ test, we assumed the change in effects across the time points (reported in Table 4) was statistically significant.

Given the six tests, two of the comparisons indicated a statistically significant change from the more parsimonious model to the model with freely estimated parameters. Neither the effects of late childhood intelligence nor socioeconomic background on income or occupational status significantly changed over time (**Hypothesis 2.1**). While we found a statistically significant increase in the effect of education on occupational status, the effect of education on income did not change over time (**Hypothesis 2.2**), partially confirming **Hypothesis 2.2** on the effect of education remaining stable over time.

### 3.3. Path Models

In the last step, we focused on whether foregoing income and occupational status completely predicted later occupational status and income, whether earlier career changes mediated the effects of late childhood intelligence, socioeconomic status, and education, or whether the latter had remaining unique effects on income and occupational status at time points beyond earlier career stages (Question 3). Table 6 shows the results for multivariate regressions of income and occupational status on autoregressive paths.^3^ The values for β represent the standardized effects of the predictors (listed in the left column) on the outcomes (listed in the top row). The effects listed as autoregressive paths can be interpreted similarly, as shown in Section 3.1. With a mean pre-tax monthly income of about 2390 euros at age 30 (logarithmized value = 7.78; see Table 1), a higher income at job entry by one SD would indicate about an additional 860 euros income at age 30. Therefore, the effect of β = 0.47 represented a difference of 36% at age 30 per SD income at job entry.

Results of the baseline autoregressive path model (Table 6) show that prior occupational status and income were strong predictors for later income (age 30: β = 0.47, age 40: β = 0.65) and occupational status (age 30: β = 0.76, age 40: β = 0.79).

Table 7 informs about the multivariate regressions of income and occupational status on childhood intelligence, socioeconomic background, education as a mediator, and autoregressive paths (cf. Figure 3). All cells, except for the rows for *R*^2^, contain the standardized effects (β) of the predictors (listed in the left column) on the outcomes (listed in the top row) at the distinctive time points (left column). Even in these extended models, which take late childhood intelligence, socioeconomic background, and quantity and quality of education (Table 7) into account, the autoregressive path effects remain the strongest predictors of income (age 30: β = 0.37, age 40: β = 0.58) and occupational status (age 30: β = 0.59, age 40: β = 0.54).^4^

However, prior occupational success did not entirely explain later success, as statistically significant effects of quantity of education remained for both income and occupational status at all time points. In addition, the quality of general education had statistically significant effects at age 30 and 40 on income but not on occupational status. Compared with the regression models without path effects, the standardized effects of education on both income and occupational status were smaller. Socioeconomic background also has a minimal unique effect on occupational status at age 30 (β = 0.05). Based on our findings; we partially dismissed Hypothesis 3.1 that all predictors have unique effects on both income and occupational status at the time points beyond job entry.

We again used chi-square difference tests (ꭓ^2^ test) to test whether the effects change or remain stable over time. Table 8 shows the results of the ꭓ^2^ tests for changes in effects of intelligence, socioeconomic background, quantity and quality of education, and autoregressive paths for predicting income and occupational status across time in the mediation models shown in Table 7. Values in Table 8 should be interpreted in the same way as in Table 5: a *p*-value > 0.05 shows that the change in effects across time that was implied in Table 7 was statistically significant. In our path models, we found a statistically significant decrease in the effect of education on occupational status over time. While this again confirmed Hypothesis 2.2 that there would be no change or even a decline in the effects of education over time, education remained the only significant predictor at the later time points. Overall, we found no changes in the effects on income over time.

## 4. Discussion

In this study, we investigated the differential importance of late childhood intelligence, socioeconomic background, and education for occupational success across the lifespan and particularly the extent to which these effects change over time. We also tested the extent to which individuals’ later careers can be predicted by their initial status at job entry or whether other predictors (intelligence, socioeconomic background, and education) remain important. The results show that both late childhood intelligence and socioeconomic background predict occupational status and income in adulthood. However, education is the strongest predictor of income and occupational status and mediates virtually all of the effects of intelligence and socioeconomic background. The effect pattern remains relatively homogenous across a career. Furthermore, the effect of education is the only unique factor beyond earlier career status. Once earlier career stages are controlled for, the unique effects of education on income do not decrease over time but are persistently significant, even at the age of 40 and far into working life.

Before discussing our results, it is important to note that even small differences in the logarithmized income and occupational status values can have a significant impact on an individual’s life. For example, a value of 7.34 as compared with a value of 7.78 in the logarithmized monthly pre-tax income (the difference between the mean at job entry and at age 30, see Table 1) corresponds to an increase from 1540 to 2392 euros in monthly pre-tax income. A change in ISEI-coded occupational status from 57 to 60 would mean a change from a commercial agent or legal clerk to a human resources consultant (see Ganzeboom and Treiman 2003). Similarly, even small or medium effects can have a great impact on an individual life. The difference of 200 euros pre-tax income per SD childhood intelligence at job entry^5^ amounts to 2400 euros per annum—about one and a half times the mean monthly pre-tax income.

### 4.1. Education as the Main Predictor of Income and Occupational Status

Our findings that late childhood intelligence and socioeconomic background correlate with and predict later occupational status and income at all career stages are in line with previous international research (Johnson et al. 1983; Brown et al. 2021; Hasl et al. 2019). In terms of predicting occupational status and income, childhood intelligence shows greater effects than socioeconomic background. These results are in line with those from other continental European countries (Sorjonen et al. 2012; Strenze 2007) and some findings from the UK (Spengler et al. 2018), but they differ from many other findings from the UK and the US, where the predictive power of socioeconomic background is equal to or even surpasses the predictive power of intelligence (Deary et al. 2005; Dubow et al. 2006; Rumberger 2010; Schoon 2008; Zax and Rees 2002).

As additional predictors, the quantity and quality of education mediate the effects of the other predictors and have the largest effect. This is in line with previous findings; Zax and Rees (2002) showed that education overtakes the effects of cognitive abilities on later income. Similarly, von Stumm et al. (2010) and Becker et al. (2019) found that education is the core mediator that mediates all effects between socioeconomic background, cognitive abilities, and later occupational outcomes. Spengler et al. (2015) found socioeconomic background to be less predictive (but still statistically significant) when education was included. Our findings are consistent with both human capital theory and the Wisconsin model of status attainment. Childhood intelligence, socioeconomic background, and education have an impact on later occupational success, and education is a key link between childhood intelligence, socioeconomic background, and later success.

Our data stem from Germany, a country where vocational education leads to highly specialized educational certificates. Our finding that education is the strongest predictor of job entry is in line with Bills (2003); it is likely that those specialized certificates assist in making good matches between employers and employees. We hypothesized that education would be a key factor in successfully obtaining a job but that cognitive abilities should have a greater effect on subsequent progression at work, with education losing its importance. Our findings do not support this hypothesis; the persistent predictive power of education across later life and the decline in the effects of cognitive abilities indicate that education accurately represents the skills, abilities, and knowledge needed to perform at work. The filtering according to educational signals at job entry leads to good fits between employers and employees. However, even later in a career, educational signals remain important, as further changes in occupational position and pay are mainly affected by education. This finding means Germany differs from many other countries with less specific certificates, such as the UK and USA. In line with Chevalier (2021) and Liu and Esteve (2021), in those countries, educational certificates seem to be less informative regarding skills and abilities, which is reflected in a rougher transition from education to work life. Bol and van de Werfhorst (2013) and Ganzach (2011) showed that cognitive abilities have persistent predictive power for income and occupational status in countries with less specialized educational certificates. In countries with highly specialized certificates, this is replaced and mediated by education.

### 4.2. Change Across Time

Second, we addressed the change over time in the predictive power of late childhood intelligence, socioeconomic background, and education. Our findings that there is no change in the effects of childhood intelligence and socioeconomic background are not in line with Ganzach (2011), who reported an increase in the importance of intelligence over time. This is likely due to a better matching in the filtering process at job entry in Germany. As Solga (2012) and Bol and van de Werfhorst (2013) have shown, education distributes occupational positions and further development, and in Germany, the certificates are relatively specific. In line with signal and filter theory, education is the single most important predictor of income and occupational status across all time points, with stable effects on income and even statistically significant increasing effects on occupational status over time.

These results are in line with findings from recent lifespan research (see Becker et al. 2019; Hasl et al. 2022; Karlson and Birkelund 2019) and underline the pivotal role of education as a mediator between childhood intelligence, socioeconomic background, and later job progression in Germany. Because of Germany’s track system, we attribute the role of education (as the main mediator to educational success) to its origins in childhood intelligence (especially in Germany). In this way, education itself contains the effects of cognitive abilities on later occupational status and income. Therefore, the pattern of education’s relatively stable effects on income and even increasing effects on occupational status reflects the hypothesized effects of intelligence observed in countries with less specialized educational certificates (Ganzach 2011).

### 4.3. Unique Effects on Income and Occupational Status After Job Entry

Finally, we checked how much previous income and status predict later income and status and if, in addition to these autoregressive career paths, the other predictors have unique effects on income and status. In line with Ng et al. (2005) and Shambrook et al. (2011), previous income and status (i.e., work experience) are the strongest predictors of later occupational success. In addition to the autoregressive development of occupational status and income, education shows persistent unique effects at later time points. Generally, the matching that occurs in Germany’s filtering process tends to lead to a good fit between employee and career (see also Bol and van der Werfhorst 2013). In our results, the allocation of new entrants to certain jobs and further careers is good but not perfect, as some corrections in later career paths occur. On the one hand, these findings support reasoning based on the filter/signal theory, as education is highly relevant for entering the labor market and remains predictive for later corrections, underscoring the importance of education in Germany. On the other hand, according to the theory of social closure (Weber [1921] 1980), the persistent importance of education could be a limiting factor for those who perform well at work but are not successful in gaining educational credentials. More open systems may be better, for instance, by allowing corrections based on performance indicators beyond education.

### 4.4. Practical Implications

In modern societies, merit theory promises that good performance leads to success. Therefore, these results should be assessed on the assumption that every effect on later success from sources other than cognitive abilities accumulated directly or indirectly by education should be seen as a threat to the merit promise (Daniels 1978). Various models have shown some effects of socioeconomic background, but the effects are rather small, not systematically found across income and occupational status, and not persistent over time. However, the findings indicate that there is something more than just performance and experience that impact our success and, as the OECD repeatedly pointed out in their “Trends Shaping Education” series, policy makers should not ignore inequality of educational opportunities for success (OECD 2013, 2016b, 2019). Given the key role of education and its important impact on later life, policies should aim to minimize the influence of socioeconomic advantage, at least when it is a unique effect and not confounded with other competencies. Our findings suggest that in countries with highly specialized certificates, such as Germany, education is the core path to economic success; this holds for children from different socioeconomic backgrounds. This might be relevant to the current international policy agenda of integrating social-emotional learning in the classroom (OECD 2024). Education enhances both students’ cognitive abilities and noncognitive abilities (e.g., self-efficacy and collaboration). Scholars have also underscored how those noncognitive abilities matter for occupational success (Cunha et al. 2006). Our results show that, at least in countries with highly specialized certificates, integrating social-emotional learning in the classroom might be a meaningful investment for policymakers to reduce social inequality.

### 4.5. Strengths and Limitations

The main strengths of this study are its extensive longitudinal data from the BIJU study and the substantial sample sizes, even in the most recent waves. The information used in this study spans the seventh grade in school to the age of 40, offering a unique perspective on occupational success and its development from job entry to later career stages. Most previous studies have either focused separately on intelligence, socioeconomic background, or education as predictors (Anger and Heineck 2006; Gutman and Schoon 2016) or on the prediction of one time point in later work life (Damian et al. 2016; Strenze 2007). Based on recent findings (Becker et al. 2019), we modeled the quantity and quality of education as a mediator of intelligence, socioeconomic background, and later income and occupational status. Having multiple indicators for late childhood intelligence and socioeconomic background allowed us to use a latent variable modeling approach.

Nevertheless, as in all studies, some limitations have to be considered. First, these data stem from just four of the sixteen German federal states (Berlin, Mecklenburg-Western Pomerania, North Rhine–Westphalia, and Saxony–Anhalt) and may, therefore, not be representative of Germany in general. The effects of the federal states were controlled for in all reported models. Since the educational system in Germany is structured federally, the extent to which the results generalize to the federal level remains an open question. For the cohort that we analyzed, no further dataset connecting youth and adulthood is available. However, in the future, some further evidence could be obtained for later cohorts with data from the National Educational Panel Study (NEPS; Blossfeld et al. 2011).

Second, because BIJU data were collected over nearly 25 years, this study suffered participation dropouts comparable to other large-scale longitudinal studies (see: Spengler et al. 2015; Stumm et al. 2010). Over time, more female participants responded (Becker et al. 2019). We controlled for gender by separately estimating our models in a female/male multi-group analysis. The main patterns of autoregressive paths and unique effects of education on occupational success remain, even at later time points (see Appendix A, Table A1). In the female subsample, we found some stronger effects of socioeconomic background and quality of education. These differential effects could be related to differential influences in male and female careers—e.g., by occupational sectors, family responsibilities, differential investment in specific domains, or general gender roles—but further inquiry is required. Nevertheless, in our sample, dropout is slightly positively selective for participants with high scores of socioeconomic background and cognitive abilities. For this reason, our data may underrepresent male participants who are less educated and have lower incomes.

Third, while our data covers key stages of the individual lifespan, such as educational success and transition into the labor market, it only reaches the age of 40. Further changes in income and occupational status may still occur after our last time point. Especially highly educated people with late entry into the labor market could further improve their salary and occupational status in later life by achieving positions that are highly prized but seldom achieved and restricted to holders of particular credentials—e.g., professorships in academia. The reported unique effects of education could even increase at later time points.

Fourth, the information used for our latent measurement of childhood intelligence stems from a rather short period of time in late childhood. While Bottenhorn et al. (2021) found evidence that intelligence is stable across adulthood, some findings point out that intelligence may not be a completely stable trait (Deary et al. 2013). While our dataset did not allow us to accomplish this, further investigations on the prediction of later occupational success could consider information on cognitive abilities at all possible time points, at least while the participants are in education.

Last, occupational success was partly explained by intelligence, socioeconomic background, and education, while the reported models explain between 0.14 and 0.51 of the variances in income (which is moderate to strong according to Cohen 1988) and between 0.43 and 0.65 of the variances in occupational status (which is strong according to Cohen 1988, there is still substantial variance in both dimensions of occupational success left unexplained by intelligence, socioeconomic background, education and autoregressive progression (see Table 7)). One important aspect that could further explain occupational success could be labor market segregation (Holbrow 2022). For example, working in research and development is rewarded differently in Science, Technology, Engineering and Mathematics (STEM) related occupational sectors (such as mechanical engineering) to sectors related to humanities (such as linguistic research). In addition, labor market dynamics could further have an effect on occupational success (Cheng 2014). For example, parenthood and parental leave can restrict the acquisition of work experience and thus restrict salary development (Sieppi and Pehkonen 2019). Discrimination in the labor market could also affect the mediating role of education, e.g., through different assessments of the same educational certificates for men and women.

## 5. Conclusions and Outlook

Our research contributes to the lifespan perspective on education and differential development of occupational success, covering central questions on intelligence, social background, and, ultimately, meritocracy in general. By using data from a highly structured country and across multiple time points in work life, we have expanded research to a national and temporal context that has received little attention. As we used measurements of childhood intelligence, socioeconomic background, and education that aim to be independent of national contexts, the results are highly comparable with results from other countries. Our findings are partially in line with meritocratic claims, as above all, intelligence—along with the associated educational success and prior occupational status—explains later occupational success. Addressing the importance of cognitive abilities and socioeconomic background for development in later life, our findings, based on latent models of childhood intelligence and socioeconomic background, depict Germany as a more intelligence-driven system. These findings contrast many other findings, mainly from the US (e.g., Dubow et al. 2006; Judge et al. 2010; Zax and Rees 2002). The pattern we found in Germany resembles more meritocratic intelligence patterns found in Northern European countries (see, e.g., Betthäuser et al. 2021; Sorjonen et al. 2012), which are considered to be more egalitarian countries. This is a sharp contrast to studies covering schooling—such as the Programme for International Student Assessment (PISA; OECD 2016a) or Progress in International Reading Literacy Study (PIRLS/IGLU; Bos et al. 2007; McElvany et al. 2023)—in which Germany tends to be seen as a rather unjust system (see, e.g., Chmielewski and Reardon 2016). Bol and van de Werfhorst (2013) conducted an international comparative study of school and transition issues indicating something similar—school and life-span indicators can substantially differ (see also Cortina 2015).

Furthermore, the German vocational educational system is unique in producing highly specific certificates that ease the transition into the labor market; this is also reflected in the rather high mediation via education. As the Programme for the International Assessment of Adult Competencies (PIAAC; Rammstedt et al. 2013) indicates, it could be very informative to compare our findings directly to countries with less specific vocational education (see also Bol and van de Werfhorst 2013). We would assume that education has lower predictive power and serves less as a mediator in those countries and that cognitive abilities and potentially social background have higher direct predictive power.

Overall, since studies such as PIAAC or PISA mainly focus on work life or school years, it is desirable for further longitudinal studies to consider the whole life course, from school years to the transition into tertiary education and job entry, and onward to further development in later (working) life. This would offer a necessary view on the long-term educational effects, not only on occupational success but on individual development, further shaping our understanding of the importance of intelligence and the emergence of social disparities across the lifespan in meritocratic societies. In addition, investigating the influence of labor market conditions on differential developments of occupational success could expand the reported results in a meaningful way and further disentangle the mediating role of education across childhood, youth, and later work life and its long-term effects. Our study provides further evidence of the importance of a lifespan perspective in developing a holistic picture of the complex interplay and variation of the individual with their personal endowments in different developmental systems.

## Figures and Tables

**Table 1:** Sociodemographic information for the total sample and by gender (female-male).

	Total (*n* = 4387)	Female (*n* = 2730)	Male (*n* = 1644)
	*n* (%)	*n* (%)	*n* (%)
Region			
West	2291 (52.4)	1393 (0.51)	898 (0.55)
			
	**Mean (SD)**	**Mean (SD)**	**Mean (SD)**
Education			
Educational Years	16.25 (2.81)	16.21 (2.78)	16.33 (2.86)
GPA educational diploma	4.58 (0.81)	4.58 (0.80)	4.57 (0.82)
GPA vocational diploma	3.22 (1.10)	3.18 (1.09)	3.29 (1.11)
Parental indicators			
Occupational status (father)	46.67 (13.08)		
Occupational status (mother)	46.52 (12.47)		
Income			
Job entry	7.34 (0.59)	7.29 (0.57)	7.43 (0.60)
Age 30	7.78 (0.64)	7.67 (0.63)	7.95 (0.61)
Age 40	8.05 (0.73)	7.87 (0.71)	8.33 (0.68)
Occupational status			
Job entry	56.66 (19.11)	56.54 (18.10)	56.88 (20.77)
Age 30	59.78 (18.78)	59.33 (18.15)	60.53 (19.80)
Age 40	59.69 (18.49)	59.15 (18.12)	60.67 (19.14)
			
	**(SD)**	**ΔMean(f.-m.) (SD)**	**(SD)**
Latent measures			
Childhood intelligence	(2.11)	−0.21 (2.10)	(2.09)
Socioeconomic Background	(7.07)	−1.48 (7.07)	(6.98)

Note. West = federal states of former Western Germany. Educational Years = years of education, both general and vocational, coded according to the CASMIN Index. GPA educational diploma = grade point average of highest certificate in general education, rotated data with 6 as best grade. GPA vocational diploma = grade point average of highest certificate in vocational education, rotated data with 6 as best grade. Occupational status = coded according to ISEI-Index. Income = logarithmic transformation of monthly gross income. Occupational status (father/mother) = coded according to the Treiman index (SIOPS). ΔMean(f.-m.) = difference in means of latent measures with means of male participants as reference.

**Table 2:** Bivariate correlations incl. latent constructs (childhood intelligence and socioeconomic background).

Constructs/Outcomes	1	2	3	4	5	6	7	8	9	10	11
1. Childhood intelligence											
2. Socioeconomic background	0.37 *										
3. Income at job entry	0.28 *	0.17 *									
4. Income at age 30	0.26 *	0.16 *	0.48 *								
5. income at age 40	0.19 *	0.22 *	0.36 *	0.68 *							
6. Occ. status at job entry	0.53 *	0.41 *	0.33 *	0.28 *	0.24 *						
7. Occ. status at age 30	0.49 *	0.41 *	0.30 *	0.34 *	0.31 *	0.76 *					
8. Occ. status at age 40	0.51 *	0.38 *	0.32 *	0.39 *	0.41 *	0.67 *	0.76 *				
9. Educational years	0.62 *	0.43 *	0.35 *	0.35 *	0.35 *	0.60 *	0.64 *	0.67 *			
10. GPA general education	0.38 *	0.21 *	0.16 *	0.19 *	0.19 *	0.25 *	0.23 *	0.27 *	0.23 *		
11. GPA vocational education	0.36 *	0.22 *	0.26 *	0.21 *	0.19 *	0.23 *	0.28 *	0.32 *	0.39 *	0.29 *	
12. gender (1 = female)	−0.06	−0.10 *	−0.12 *	−0.27 *	−0.33 *	0.05	−0.02	−0.02	−0.07 *	0.02	−0.01

Note. Childhood intelligence = late childhood intelligence measured by two subdimensions of KFT and IST-test, latently modeled. Socioeconomic background = socioeconomic background measured by parental occupational status and education, latently modeled. Income = logarithmic transformation of monthly gross income. Occ. status = occupational status coded according to ISEI-Index. Years of education = years of education, both general and vocational, coded according to CASMIN-Index. GPA general education = GPA of the highest educational certificate. GPA vocational education = GPA of highest vocational certificates. * *p* < 0.05.

**Table 3:** Multivariate regressions of income and occupational status on childhood intelligence and socioeconomic background.

	Income			Occ. Status		
	β	[95% CI]		β	[95% CI]	
		Lower	Upper		Lower	Upper
Job entry						
Child. intelligence	0.21 *	0.12	0.30	0.42 *	0.35	0.48
Soc. background	0.12 *	0.02	0.21	0.25 *	0.18	0.31
*R* ^2^	0.10			0.32		
Age 30						
Child. intelligence	0.21 *	0.13	0.30	0.38 *	0.31	0.46
Soc. background	0.09 *	0.01	0.17	0.27 *	0.19	0.34
*R* ^2^	0.08			0.30		
Age 40						
Child. intelligence	0.12	−0.01	0.23	0.41 *	0.32	0.50
Soc. background	0.17 *	0.09	0.25	0.22 *	0.13	0.31
*R* ^2^	0.06			0.29		

Note. Child. intelligence = late childhood intelligence measured by two sub dimensions of KFT and IST test, latently modeled. Soc. background = socioeconomic background measured by parental occupational status and education, latently modeled. Occ. status = occupational status coded according to ISEI-Index. Income = logarithmic transformation of monthly gross income. * *p* < 0.05.

**Table 4:** Multivariate regressions of income and occupational status on childhood intelligence, socioeconomic background, and education as a mediator.

	Education			Income	Occ. Status
	Education (Years)	GPA gen. edu.	GPA voc. edu.		
Education					
Child. intelligence	0.54 *	0.38 *	0.41 *		
Soc. background	0.24 *	0.05	0.07		
Job entry					
Child. intelligence				0.05	0.25 *
Soc. background				0.03	0.17 *
Education years				0.24 *	0.39 *
GPA gen. edu.				0.06	0.06
GPA voc. edu.				0.06	−0.10 *
*R* ^2^				0.13	0.42
Age 30					
Child. intelligence				−0.01	0.11 *
Soc. background				−0.01	0.15 *
Education years				0.28 *	0.50 *
GPA gen. edu.				0.12 *	0.04
GPA voc. edu.				0.06	−0.01
*R* ^2^				0.13	0.45
Age 40					
Child. intelligence				−0.13	0.09
Soc. background				0.06	0.09 *
Education years				0.32 *	0.55 *
GPA gen. edu.				0.15 *	0.07 *
GPA voc. edu.				0.06	0.03
*R* ^2^				0.14	0.48

Note. Values represent standardized effects (β). Child. intelligence = Late childhood intelligence measured by two sub dimensions of KFT and IST test, latently modeled. Soc. Background = Socioeconomic Background measured by parental occupational status and education, latently modeled. Occ. status = occupational status coded according to the ISEI Index. Income = Logarithmic transformation of monthly gross income. Education years = Years of education, both general and vocational. GPA gen. edu. = GPA of the highest educational certificate. GPA voc. edu. = GPA of highest vocational certificates. * *p* < 0.05.

**Table 5:** ꭓ^2^ tests for changes in the effects of intelligence, socioeconomic background, and quantity and quality of education for predicting income and occupational status across time.

	β Estimated Freely (Reference Model)			β Constrained to Be Equal			*p*-Value
	ꭓ^2^ Value	*df*	Scaling Factor	ꭓ^2^ Value	*df*	Scaling Factor	
Income							
Reference Model	288.06	66	2.37				
Test on parameter equality for…							
Child. intelligence				292.01	68	2.36	0.20
Soc. background				291.01	68	2.38	0.21
Education years				294.02	68	2.37	0.06
GPA gen. edu.				293.58	68	2.36	0.09
GPA voc. edu.				285.53	68	2.39	0.09
							
Occupational Status							
Reference Model	260.20	66	2.51				
Test on parameter equality for…							
Child. intelligence				265.74	68	2.52	0.06
Soc. background				262.38	68	2.52	0.21
Education years				281.30	68	2.50	<0.01 *
GPA gen. edu.				261.43	68	2.51	0.58
GPA voc. edu.				273.12	68	2.51	<0.01 *

Note. Reference model: all parameters freely estimated. Child. intelligence = late childhood intelligence measured by two sub dimensions of KFT and IST test, latently modeled. Soc. background = socioeconomic background measured by parental occupational status and education, latently modeled. Occupational status = occupational status coded according to ISEI-Index. Income = logarithmic transformation of monthly gross income. Education years = years of education, both general and vocational. GPA gen. edu. = GPA of the highest educational certificate. GPA voc. edu. = GPA of highest vocational certificates. * *p* < 0.05.

**Table 6:** Multivariate regressions of income and occupational status on autoregressive paths.

	Income			Occupational Status		
	β	[95% CI]		β	[95% CI]	
		Lower	Upper		Lower	Upper
Autoregressive paths						
Age 30 on job entry	0.47 *	0.41	0.53	0.76 *	0.73	0.79
*R* ^2^	0.22			0.58		
Age 40 on age 30	0.65 *	0.59	0.71	0.79 *	0.75	0.83
*R* ^2^	0.42			0.62		

Note. * *p* < .05.

**Table 7:** Multivariate regressions of income and occupational status on late childhood intelligence, socioeconomic background, education as a mediator, and autoregressive paths.

	Education			Income	Occ. Status
	Education (Years)	GPA gen. edu.	GPA voc. edu.		
Education					
Child. intelligence	0.54 *	0.38 *	0.41 *		
Soc. background	0.24 *	0.05	0.07		
Job entry					
Child. intelligence				0.05	0.26 *
Soc. Background				0.03	0.18 *
Years in education				0.25 *	0.37 *
GPA gen. edu.				0.06	0.05
GPA voc. edu.				0.07	−0.10 *
*R* ^2^				0.14	0.43
Age 30					
Child. intelligence				−0.01	−0.04
Soc. background				−0.02	0.05 *
Education years				0.18 *	0.28 *
GPA gen. edu.				0.10 *	0.01
GPA voc. edu.				0.03	0.05
Autoregressive path				0.37 *	0.59 *
*R* ^2^				0.32	0.64
Age 40					
Child. intelligence				−0.13	0.08
Soc. background				0.07	0.02
Education years				0.16 *	0.26 *
GPA gen. edu.				0.07 *	0.04
GPA voc. edu.				0.03	0.02
Autoregressive path				0.58 *	0.54 *
*R* ^2^				0.51	0.65

Note. Values represent standardized effects (β). Child. intelligence = Late childhood intelligence measured by two subdimensions of KFT and IST test, latently modeled. Soc. Background = Socioeconomic Background measured by parental occupational status and education, latently modeled. Occ. status = occupational status coded according to the ISEI Index. Income = Logarithmic transformation of monthly gross income. Education (years) = Years of education, both general and vocational. GPA gen. edu. = GPA of the highest educational certificate. GPA voc. edu. = GPA of highest vocational certificates. * *p* < 0.05.

**Table 8:** ꭓ^2^ tests for changes in effects of intelligence, socioeconomic background, quantity and quality of education, and autoregressive paths for predicting income and occupational status across time.

	β Estimated Freely (Reference Model)			β Constrained to Be Equal			*p*-Value
	ꭓ^2^ Value	*df*	Scaling Factor	ꭓ^2^ Value	*df*	Scaling Factor	
Income							
Reference model	290.23	67	2.36				
Test on parameter equality for…							
Child. intelligence				294.20	69	2.35	0.20
Soc. background				291.84	69	2.37	0.21
Education years				290.82	69	2.36	0.64
GPA gen. edu.				291.86	69	2.35	0.70
GPA voc. edu.				289.09	69	2.38	0.74
							
Occupational Status							
Reference Model	272.40	67	2.52				
Test on parameter equality for…							
Child. intelligence				286.57	69	2.55	>0.01 *
Soc. background				285.52	69	2.52	>0.01 *
Education years				269.65	69	2.60	0.27
GPA gen. edu.				273.04	69	2.53	0.51
GPA voc. edu.				285.20	69	2.54	>0.01 *

Note. Reference model: all parameters freely estimated. Child. intelligence = late childhood intelligence measured by two sub dimensions of KFT and IST test, latently modeled. Soc. background = socioeconomic background measured by parental occupational status and education, latently modeled. Occupational status = occupational status coded according to ISEI-Index. Income = logarithmic transformation of monthly gross income. Education years = years of education, both general and vocational. GPA gen. edu. = GPA of the highest educational certificate. GPA voc. edu. = GPA of highest vocational certificates. * *p* < .05.

## Data Availability

An anonymized version of key data presented in this study will be available upon request from the principal investigator of this study https://www.dipf.de/en/research/projects/learning-processes-educational-careers-and-psychosocial-development-in-adolescence-and-young-adulthood-study-biju (accessed on 4 March 2025). We are not in a position to make data publicly available because these contain information that could compromise research participants’ privacy and consent.

## Notes

1 A detailed description of dropout and sample selectivity can be found in Becker et al. (2019).

2 For an interpretation of changes in logarithmized income and ISEI-coded occupational status, see Section 4. “Discussion”. For an interpretation of changes in the effects, see Section 3.1. “Predicting Income and Occupational Status at Different Stages in Work Life”.

3 The autoregressive paths are shown in Figure 3 as the paths that connect income/occupational status at job entry with income/occupational status at age 30 and income/occupational status at age 30 with income/occupational status at age 40. Table 6 contains informs about a model in which just the autoregressive paths and no other predictors are estimated.

4 For results in a multivariate regression of income and occupational status on autoregressive paths, late childhood intelligence, and socioeconomic background without education as a mediator, see Table A2 in Appendix A.

5 See Section 3.1. “Predicting Income and Occupational Status at Different Stages in Work Life”.

## Supplementary Materials

The supporting information can be downloaded at: https://www.mdpi.com/article/10.3390/jintelligence13030032/s1, Supplementary Information: MPLUS Syntax used.

## Appendix A

**Table A1:** Multivariate regressions of income and occupational status on socioeconomic background, cognitive abilities, education as a mediator, and autoregressive paths, separated by gender.

	Male					Female				
	Education			Income	Occ. Status	Education			Income	Occ. Status
	Education (Years)	GPA gen. edu.	GPA voc. edu.			Education (Years)	GPA gen. edu.	GPA voc. edu.		
Education										
Child. intelligence	0.56 *	0.47 *	0.45 *			0.53 *	0.34 *	0.40 *		
Soc. background	0.26 *	−0.06	−0.04			0.23 *	0.11 *	0.10 *		
Job entry										
Child. intelligence				0.03	0.30 *				0.06	0.25 *
Soc. background				−0.01	0.17 *				0.06	0.15 *
Education years				0.34 *	0.40 *				0.19 *	0.36 *
GPA gen. edu.				0.01	−0.01				0.09 *	0.10 *
GPA voc. edu.				0.10	−0.11				0.05 *	−0.09 *
*R* ^2^				0.18	0.49				0.13	0.59
Age 30										
Child. intelligence				−0.07	−0.10				0.02	0.00
Soc. background				−0.04	0.06 *				−0.01	0.04
Education years				0.16	0.28 *				0.21 *	0.28 *
GPA gen. edu.				0.13	−0.01				0.08	0.03
GPA voc. edu.				0.10	0.10 *				0.00	0.01
Autoregressive path				0.40 *	0.61 *				0.38 *	0.57 *
*R* ^2^				0.28	0.66				0.26	0.37
Age 40										
Child. intelligence				0.01	0.16				−0.14	0.05
Soc. background				−0.02	−0.02				0.09 *	0.04
Education years				0.15	0.34 *				0.17 *	0.20 *
GPA gen. edu.				0.02	−0.01				0.09	0.04
GPA voc. edu.				−0.03	−0.06				0.03	0.07
Autoregressive path				0.68 *	0.47 *				0.53 *	0.58 *
*R* ^2^				0.55	0.68				0.39	0.36

Note. Values represent standardized effects (β). Child. intelligence = Late childhood intelligence measured by two sub dimensions of KFT and IST test, latently modeled. Soc. Background = Socioeconomic Background measured by parental occupational status and education, latently modeled. Occ. status = occupational status coded according to ISEI-Index. Income = Logarithmic transformation of monthly gross income. Education (years) = Years of education, both general and vocational. GPA gen. edu. = GPA of the highest educational certificate. GPA voc. edu. = GPA of highest vocational certificates. * *p* < 0.05.

**Table A2:** Stepwise multivariate regressions of income and occupational status on (1) autoregressive paths, (2) late childhood intelligence and socioeconomic background, and (3) education as a mediator.

	Education			Income			Occupational Status		
	Education (Years)	GPA gen. edu.	GPA voc. edu.	Path Only (1)	Without Mediation (2)	With Mediation (3)	Path Only (1)	Without Mediation (2)	With Mediation (3)
Education									
Child. intelligence	0.54 *	0.38 *	0.41 *						
Soc. background	0.24 *	0.05	0.07						
Job entry									
Child. intelligence					0.21 *	0.05		0.44 *	0.26 *
Soc. background					0.12 *	0.03		0.25 *	0.18 *
Education years						0.25 *			0.37 *
GPA gen. edu.						0.06			0.05
GPA voc. edu.						0.07			−0.10 *
*R* ^2^					0.10	0.14		0.32	0.43
Age 30									
Child. intelligence					0.12 *	−0.01		0.08 *	−0.04
Soc. background					−0.04	−0.02		0.10 *	0.05 *
Education years						0.18 *			0.28 *
GPA gen. edu.						0.10 *			0.01
GPA voc. edu.						0.03			0.05
Autoregressive path				0.47 *	0.44 *	0.37 *	0.76 *	0.67 *	0.59 *
*R* ^2^				0.22	0.25	0.32	0.58	0.59	0.64
Age 40									
Child. intelligence					−0.02	−0.13		0.20 *	0.08
Soc. background					0.11 *	0.07		0.04	0.02
Education years						0.16 *			0.26 *
GPA gen. edu.						0.07 *			0.04
GPA voc. edu.						0.03			0.02
Autoregressive path				0.65 *	0.67 *	0.58 *	0.79 *	0.65 *	0.54 *
*R* ^2^				0.42	0.48	0.51	0.61	0.62	0.65

Note. Values represent standardized effects (β). Child. intelligence = Late childhood intelligence measured by two sub dimensions of KFT and IST test, latently modeled. Soc. background = Socioeconomic Background measured by parental occupational status and education, latently modeled. Occ. status = occupational status coded according to ISEI-Index. Income = Logarithmic transformation of monthly gross income. Education (years) = Years of education, both general and vocational. GPA gen. edu. = GPA of the highest educational certificate. GPA voc. edu. = GPA of highest vocational certificates. * *p* < 0.05.

## References

[B1-jintelligence-13-00032] Altonji Joseph G., Pierret Charles R. (2001). Employer Learning and Statistical Discrimination. The Quarterly Journal of Economics.

[B2-jintelligence-13-00032] Amthauer Rudolf (1955). I-S-T. Intelligenz-Struktur-Test: Handanweisung für die Durchführung und Auswertung [Intelligence Structure Test: Manual for Administration and Analysis].

[B3-jintelligence-13-00032] Amthauer Rudolf, Brocke Burkhard, Liepmann Detlev, Beauducel André (2001). Intelligenz-Struktur-Test 2000 R [Intelligence Structure Test 2000 R].

[B4-jintelligence-13-00032] Anger Silke, Heineck Guido (2006). Cognitive Abilities and Labour Market Outcomes: First Evidence for Germany.

[B5-jintelligence-13-00032] Arrow Kenneth J. (1973). Higher education as a filter. Journal of Public Economics.

[B6-jintelligence-13-00032] Baltes Paul B. (1987). Theoretical propositions of life-span developmental psychology: On the dynamics between growth and decline. Developmental Psychology.

[B7-jintelligence-13-00032] Banaszczuk Yasmina, Banaszczuk Yasmina (2017). Netzwerke beim Berufseinstieg—Strukturen, Nutzungsweisen und soziale Herkunft [Networks at career entry—structures, usage patterns and social background]. Netzwerke beim Berufseinstieg: Strukturen, Nutzungsweisen und soziale Herkunft [Networks at Career Entry: Structures, Usage Patterns and Social Background].

[B8-jintelligence-13-00032] Baumert Jürgen, Roeder Peter M., Gruehn Sabine, Heyn Susanne, Köller Olaf, Rimmele Rolf, Schnabel Kai U., Seipp Bettina, Treumann Klaus-Peter, Neubauer Georg, Möller Renate, Abel Jürgen (1996). Bildungsverläufe und psychosoziale Entwicklung im Jugendalter (BIJU) [Educational Careers and Psychosocial Development in Adolescence and Young Adulthood]. Methoden und Anwendungen empirischer pädagogischer Forschung [Methods and Applications of Empirical Educational Research].

[B9-jintelligence-13-00032] Becker Gary S. (2009). Human Capital: A Theoretical and Empirical Analysis, with Special Reference to Education.

[B10-jintelligence-13-00032] Becker Michael, Tetzner Julia (2021). On the relations of sociocognitive childhood characteristics, education, and socioeconomic success in adulthood. Contemporary Educational Psychology.

[B11-jintelligence-13-00032] Becker Michael, Baumert Jürgen, Tetzner Julia, Maaz Kai, Köller Olaf (2019). Childhood Intelligence, Family Background, and Gender as Drivers of Socioeconomic Success: The Mediating Role of Education. Developmental Psychology.

[B12-jintelligence-13-00032] Belley Philippe, Lochner Lance (2007). The Changing Role of Family Income and Ability in Determining Educational Achievement. Journal of Human Capital.

[B13-jintelligence-13-00032] Bergman Lars R., Corovic Jelena, Ferrer-Wreder Laura, Modig Karin (2014). High IQ in Early Adolescence and Career Success in Adulthood: Findings from a Swedish Longitudinal Study. Research in Human Development.

[B14-jintelligence-13-00032] Bernardi Fabrizio, Boertien Diederik, Geven Koen (2019). Childhood Family Structure and the Accumulation of Wealth Across the Life Course. Journal of Marriage and the Family.

[B15-jintelligence-13-00032] Betthäuser Bastian A., Kaiser Caspar, Trinh Nhat an (2021). Regional Variation in Inequality of Educational Opportunity across Europe. Socius: Sociological Research for a Dynamic World.

[B16-jintelligence-13-00032] Bills David B. (2003). Credentials, Signals, and Screens: Explaining the Relationship Between Schooling and Job Assignment. Review of Educational Research.

[B17-jintelligence-13-00032] Bleidorn Wiebke (2012). Hitting the Road to Adulthood: Short-Term Personality Development During a Major Life Transition. Personality & Social Psychology Bulletin.

[B18-jintelligence-13-00032] Blossfeld Hans-Peter (1989). Kohortendifferenzierung und Karriereprozess: Eine Längsschnittstudie über die Veränderung der Bildungs- und Berufschancen im Lebenslauf [Cohort Differentiation and the Career Process: A Longitudinal Study on the Change in Educational and Career Opportunities over the Life Course].

[B19-jintelligence-13-00032] Blossfeld Hans-Peter, Maurice Jutta von, Roßbach Hans-Günther (2011). Education as a Lifelong Process: The German National Educational Panel Study (NEPS).

[B20-jintelligence-13-00032] Bol Thijs, van de Werfhorst Herman G. (2013). Educational Systems and the Trade-Off between Labor Market Allocation and Equality of Educational Opportunity. Comparative Education Review.

[B21-jintelligence-13-00032] Bos Wilfried, Hornberg Sabine, Arnold Karl-Heinz, Faust-Siehl Gabriele, Fried Lilian, Lankes Eva-Maria, Schwippert Knut, Tarelli Irmela, Valtin Renate (2007). IGLU 2006—die Grundschule auf dem Prüfstand: Vertiefende Analysen zu Rahmenbedingungen schulischen Lernens [IGLU 2006—Examining Elementary School: Deepening Analyses of the Conditions of Learning at School].

[B22-jintelligence-13-00032] Bottenhorn Katherine L., Bartley Jessica E., Riedel Michael C., Salo Taylor, Bravo Elsa I., Odean Rosalie, Nazareth Alina, Laird Robert W., Musser Erica D., Pruden Shannon M. (2021). Intelligence and academic performance: Is it all in your head?. bioRxiv.

[B23-jintelligence-13-00032] Bozick Robert, Alexander Karl, Entwisle Doris, Dauber Susan, Kerr Kerri (2010). Framing the Future: Revisiting the Place of Educational Expectations in Status Attainment. Social Forces.

[B24-jintelligence-13-00032] Brandt Naemi D., Israel Anne, Becker Michael, Wagner Jenny (2021). The joint power of personality and motivation dynamics for occupational success: Bridging two largely separated fields. European Journal of Personality.

[B25-jintelligence-13-00032] Brown Matt I., Wai Jonathan, Chabris Christopher F. (2021). Can You Ever Be Too Smart for Your Own Good? Comparing Linear and Nonlinear Effects of Cognitive Ability on Life Outcomes. Perspectives on Psychological Science: A Journal of the Association for Psychological Science.

[B26-jintelligence-13-00032] Brydges Christopher R. (2019). Effect Size Guidelines, Sample Size Calculations, and Statistical Power in Gerontology. Innovation in Aging.

[B27-jintelligence-13-00032] Buchmann Marlis C., Steinhoff Annekatrin (2017). Social Inequality, Life Course Transitions, and Adolescent Development: Introduction to the Special Issue. Journal of Youth and Adolescence.

[B28-jintelligence-13-00032] Buchmann Marlis C., Kriesi Irene (2011). Transition to Adulthood in Europe. Annual Review of Sociology.

[B29-jintelligence-13-00032] Bukodi Erzsébet, Bourne Mollie, Betthäuser Bastian (2017). Wastage of talent?. Advances in Life Course Research.

[B30-jintelligence-13-00032] Bukodi Erzsébet, Erikson Robert, Goldthorpe John H. (2014). The effects of social origins and cognitive ability on educational attainment. Acta Sociologica.

[B31-jintelligence-13-00032] Byington Eliza, Felps Will (2010). Why do IQ scores predict job performance?. Research in Organizational Behavior.

[B32-jintelligence-13-00032] Byrne Barbara M. (2012). Structural Equation Modeling with Mplus: Basic Concepts, Applications, and Programming.

[B33-jintelligence-13-00032] Cawley John, Heckman James, Vytlacil Edward (2001). Three observations on wages and measured cognitive ability. Labour Economics.

[B34-jintelligence-13-00032] Cheng Siwei (2014). A Life Course Trajectory Framework for Understanding the Intracohort Pattern of Wage Inequality. American Journal of Sociology.

[B35-jintelligence-13-00032] Chevalier Tom, Castrén Anna-Maija, Česnuitytė Vida, Crespi Isabella, Gauthier Jacques-Antoine, Gouveia Rita, Martin Claude, Mínguez Almudena Moreno, Suwada Katarzyn (2021). Varieties of Youth Transitions? A Review of the Comparative Literature on the Entry to Adulthood. The Palgrave Handbook of Family Sociology in Europe.

[B36-jintelligence-13-00032] Chmielewski Anna K., Reardon Sean F. (2016). Patterns of Cross-National Variation in the Association Between Income and Academic Achievement. AERA Open.

[B37-jintelligence-13-00032] Cohen Jacob (1988). Statistical Power Analysis for the Behavioral Sciences.

[B38-jintelligence-13-00032] Collins Randall, Cottom Tressie McMillan, Stevens Mitchell L. (2019). The Credential Society: An Historical Sociology of Education and Stratification: New Preface.

[B39-jintelligence-13-00032] Conley Dalton, Gifford Brian (2006). Home Ownership, Social Insurance, and the Welfare State. Sociological Forum.

[B40-jintelligence-13-00032] Cortina Kai S. (2015). PIAAC and PISA: Pedagogically Paradoxical Parallels. Zeitschrift für Pädagogik.

[B41-jintelligence-13-00032] Credé Marcus, Kuncel Nathan R. (2008). Study Habits, Skills, and Attitudes: The Third Pillar Supporting Collegiate Academic Performance. Perspectives on Psychological Science: A Journal of the Association for Psychological Science.

[B42-jintelligence-13-00032] Crouse James, Mueser Peter, Jencks Christopher, Reichardt Charles S. (1979). Latent variable models of status attainment. Social Science Research.

[B43-jintelligence-13-00032] Cunha Flavio, Heckman James J. (2007). The Technology of Skill Formation. American Economic Review.

[B44-jintelligence-13-00032] Cunha Flavio, Heckman James J., Lochner Lance, Masterov Dimitriy V., Hanushek Eric, Welch Finis (2006). Interpreting the Evidence on Life Cycle Skill Formation. Handbook of the Economics of Education Volume 1.

[B45-jintelligence-13-00032] Damian Rodica Ioana, Su Rong, Shanahan Michael, Trautwein Ulrich, Roberts Brent W. (2016). Can Personality Traits and Intelligence Compensate for Background Disadvantage? Predicting Status Attainment in Adulthood. Journal of Personality and Social Psychology.

[B46-jintelligence-13-00032] Daniels Norman (1978). Merit and meritocracy. Justice and Justification: Reflective Equilibrium in Theory and Practice.

[B47-jintelligence-13-00032] Dannefer Dale (2003). Cumulative Advantage/disadvantage and the Life Course: Cross-Fertilizing Age and Social Science Theory. The Journals of Gerontology. Series B, Psychological Sciences and Social Sciences.

[B48-jintelligence-13-00032] Davis-Kean Pamela E. (2005). The Influence of Parent Education and Family Income on Child Achievement: The Indirect Role of Parental Expectations and the Home Environment. Journal of Family Psychology: JFP: Journal of the Division of Family Psychology of the American Psychological Association (Division 43).

[B49-jintelligence-13-00032] Deary Ian J., Pattie Alison, Starr John M. (2013). The Stability of Intelligence from Age 11 to Age 90 Years: The Lothian Birth Cohort of 1921. Psychological Science.

[B50-jintelligence-13-00032] Deary Ian J., Taylor Michelle D., Hart Carole L., Wilson Valerie, Smith George Davey, Blane David, Starr John M. (2005). Intergenerational social mobility and mid-life status attainment: Influences of childhood intelligence, childhood social factors, and education. Intelligence.

[B51-jintelligence-13-00032] Deary Ian J., Strand Steve, Smith Pauline, Fernandes Cres (2007). Intelligence and educational achievement. Intelligence.

[B52-jintelligence-13-00032] Diehl Claudia, Hunkler Christian, Kristen Cornelia (2016). Ethnische Ungleichheiten im Bildungsverlauf [Ethnic Inequality in the Educational Career].

[B53-jintelligence-13-00032] DiPrete Thomas A., Eirich Gregory M. (2006). Cumulative Advantage as a Mechanism for Inequality: A Review of Theoretical and Empirical Developments. Annual Review of Sociology.

[B54-jintelligence-13-00032] DiPrete Thomas A., Bol Thijs, Eller Christina Ciocca, Werfhorst Herman G. van de (2017). School-to-Work Linkages in the United States, Germany, and France. American Journal of Sociology.

[B55-jintelligence-13-00032] Dubow Eric F., Huesmann L. Rowell, Boxer Paul, Pulkkinen Lea, Kokko Katja (2006). Middle Childhood and Adolescent Contextual and Personal Predictors of Adult Educational and Occupational Outcomes: A Mediational Model in Two Countries. Developmental Psychology.

[B56-jintelligence-13-00032] Fitzsimons Patrick, Peters Michael (2017). Human Capital Theory and Education. Encyclopedia of Educational Philosophy and Theory.

[B57-jintelligence-13-00032] Forster Andrea, Bol Thijs, Werfhorst Herman van de (2016). Vocational Education and Employment over the Life Cycle. Sociological Science.

[B58-jintelligence-13-00032] Franz Wolfgang, Franz Wolfgang (2013). Investitionen in das Humankapital. Arbeitsmarktökonomik.

[B59-jintelligence-13-00032] Fujishiro Kaori, Xu Jun, Gong Fang (2010). What Does “Occupation” Represent as an Indicator of Socioeconomic Status? Exploring Occupational Prestige and Health. Social Science & Medicine.

[B60-jintelligence-13-00032] Gangl Markus (2003). Bildung und Übergangsrisiken beim Einstieg in den Beruf [Education and transition risks when entering the world of work]. Zeitschrift für Erziehungswissenschaft.

[B61-jintelligence-13-00032] Ganzach Yoav (2011). A dynamic analysis of the effects of intelligence and socioeconomic background on job-market success. Intelligence.

[B62-jintelligence-13-00032] Ganzeboom Harry B. G. (2010). Questions and Answers About ISEI-08. http://www.harryganzeboom.nl/isco08/qa-isei-08.htm.

[B63-jintelligence-13-00032] Ganzeboom Harry B. G., Treiman Donald J., Hoffmeyer-Zlotnik Jürgen H. P., Wolf Christof (2003). Three Internationally Standardised Measures for Comparative Research on Occupational Status. Advances in Cross-National Comparison.

[B64-jintelligence-13-00032] Gecas Viktor, Mortimer Jeylan T., Shanahan Michael J. (2003). Self-Agency and the Life Course. Handbook of the Life Course.

[B65-jintelligence-13-00032] Geis Alfons J., Körber Kristina (2011). Handbuch für die Berufsvercodung. [Manual for Career Coding].

[B66-jintelligence-13-00032] Geißler Ferdinand (2018). Bildung, Fähigkeiten und Arbeitsmarkterträge [Education, Abilities and Labor Market Results].

[B67-jintelligence-13-00032] Georg Werner, Fend Helmut, Berger Fred, Grob Urs (2009). Prädiktion des Berufsstatus—Zur unterschiedlichen Bedeutung personaler Ressourcen bei Frauen und Männern [Predicting Occupational Status—The Different Significance of Personal Resources for Women and Men]. Lebensverläufe, Lebensbewältigung, Lebensglück: Ergebnisse der LifE-Studie.

[B68-jintelligence-13-00032] Giele Janet Z., Elder Glen H., Giele Janet Z., Elder Glen H. (1998). Life Course Research: Development of a Field. Methods of Life Course Research: Qualitative and Quantitative Approaches.

[B69-jintelligence-13-00032] Gottfredson Linda S., Nyborg Helmuth (2003). Chapter 15—G, Jobs and Life. The Scientific Study of General Intelligence: Tribute to Arthur Jensen.

[B70-jintelligence-13-00032] Graham John W. (2009). Missing Data Analysis: Making It Work in the Real World. Annual Review of Psychology.

[B71-jintelligence-13-00032] Gutman Leslie Morrison, Schoon Ingrid, Khine Myint S., Areepattamannil Shaljan (2016). A Synthesis of Causal Evidence Linking Non-Cognitive Skills to Later Outcomes for Children and Adolescents. Non-Cognitive Skills and Factors in Educational Attainment.

[B72-jintelligence-13-00032] Hanushek Eric A., Schwerdt Guido, Woessmann Ludger, Zhang Lei (2017). General Education, Vocational Education, and Labor-Market Outcomes over the Lifecycle. Journal of Human Resources.

[B73-jintelligence-13-00032] Hasl Andrea, Kretschmann Julia, Richter Dirk, Voelkle Manuel, Brunner Martin (2019). Investigating Core Assumptions of the “American Dream”: Historical Changes in How Adolescents’ Socioeconomic Status, IQ, and GPA Are Related to Key Life Outcomes in Adulthood. Psychology and Aging.

[B74-jintelligence-13-00032] Hasl Andrea, Voelkle Manuel, Kretschmann Julia, Richter Dirk, Brunner Martin (2022). A Dynamic Structural Equation Approach to Modeling Wage Dynamics and Cumulative Advantage Across the Lifespan. Multivariate Behavioral Research.

[B75-jintelligence-13-00032] Heckman James, Carneiro Pedro (2003). Human Capital Policy.

[B76-jintelligence-13-00032] Hegelund Emilie Rune, Flensborg-Madsen Trine, Dammeyer Jesper, Mortensen Erik Lykke (2020). The Modifying Influence of Family Social Background on the Association Between IQ and Unsuccessful Educational and Occupational Achievement. Journal of Individual Differences.

[B77-jintelligence-13-00032] Heimann Anna L., Ingold Pia V., Debus Maike E., Kleinmann Martin (2021). Who will go the extra mile? Selecting organizational citizens with a personality-based structured job interview. Journal of Business and Psychology.

[B78-jintelligence-13-00032] Heinz Walter R., Mortimer Jeylan T., Shanahan Michael J. (2003). From Work Trajectories to Negotiated Careers. Handbook of the Life Course.

[B79-jintelligence-13-00032] Heller Kurt A., Perleth Christoph (2000). Kognitiver Fähigkeitstest für 4.-12. Klassen, Revision (KFT 4–12+ R) [Cognitive Ability Test for Grades 4–12, Revision].

[B80-jintelligence-13-00032] Heller Kurt A., Schoen-Gaedike Anne-Katrin, Weinlaeder Helga (1985). Kognitiver Fähigkeitstest: KFT 4–13 [Cognitive Ability Test: KFT 4–13+].

[B81-jintelligence-13-00032] Holbrow Hilary J. (2022). When All Assistants Are Women, Are All Women Assistants? Gender Inequality and the Gender Composition of Support Roles. The Russell Sage Foundation Journal of the Social Sciences.

[B82-jintelligence-13-00032] Huntington-Klein Nick (2021). Human Capital versus signaling is empirically unresolvable. Empirical Economics.

[B83-jintelligence-13-00032] International Labour Office (2012). International Standard Classification of Occupations 2008 (ISCO-08): Structure, Group Definitions and Correspondence Tables.

[B84-jintelligence-13-00032] International Labour Organization (1990). International Standard Classification of Occupations: ISCO-88.

[B85-jintelligence-13-00032] Jacob Marita, Klein Markus (2013). Der Einfluss der Bildungsherkunft auf den Berufseinstieg und die ersten Erwerbsjahre von Universitätsabsolventen [The influence of educational background on career entry and the first years of employment of university graduates]. Beiträge zur Hochschulforschung.

[B86-jintelligence-13-00032] Johnson Ronald. C., Nagoshi Craig T., Ahem Frank M., Wilson James R., DeFries John C., McClearn Gerald E., Vandenberg Steven. G. (1983). Family Background, Cognitive Ability, and Personality as Predictors of Educational and Occupational Attainment. Social Biology.

[B87-jintelligence-13-00032] Judge Timothy A., Klinger Ryan L., Simon Lauren S. (2010). Time Is on My Side: Time, General Mental Ability, Human Capital, and Extrinsic Career Success. The Journal of Applied Psychology.

[B88-jintelligence-13-00032] Karlson Kristian Bernt, Birkelund Jesper Fels (2019). Education as a Mediator of the Association Between Origins and Destinations: The Role of Early Skills. Research in Social Stratification and Mobility.

[B89-jintelligence-13-00032] Kerckhoff Alan C., Mortimer Jeylan T., Shanahan Michael J. (2003). From Student to Worker. Handbook of the Life Course.

[B90-jintelligence-13-00032] Konietzka Dirk, Hensel Tom, Becker Rolf (2017). Berufliche Bildung im Lebenslauf. Grundlage und empirische Befunde [Vocational Training in the Life Course. Basis and Empirical Findings]. Lehrbuch der Bildungssoziologie.

[B91-jintelligence-13-00032] König Wolfgang, Lüttinger Paul, Müller Walter (1988). A Comparative Analysis of the Development and Structure of Educational Systems: Methodological Foundations and the Construction of a Comparative Educational Scale (CASMIN Working Paper No. 12).

[B92-jintelligence-13-00032] Kramer Jochen (2009). Allgemeine Intelligenz und beruflicher Erfolg in Deutschland [General Intelligence and Occupational Success in Germany]. Psychologische Rundschau.

[B93-jintelligence-13-00032] Liepmann Detlev, Beauducel André, Brocke Burkhard, Amthauer Rudolf (2007). I-S-T 2000 R: Intelligenz-Struktur-Test 2000 R [Intelligence Structure Test 2000 R].

[B94-jintelligence-13-00032] Lin Dajun, Lutter Randall, Ruhm Christopher (2016). Cognitive Performance and Labor Market Outcomes.

[B95-jintelligence-13-00032] Liu Chia, Esteve Albert, Schneider Norbert F. (2021). Living arrangements across households in Europe. Research Handbook on the Sociology of the Family.

[B96-jintelligence-13-00032] Liwiński Jacek, Pastore Francesco (2021). Are School-Provided Skills Useful at Work? Results of the Wiles Test. Research in Higher Education.

[B97-jintelligence-13-00032] Lubinski David, Benbow Camilla P., Kell Harrison J. (2014). Life Paths and Accomplishments of Mathematically Precocious Males and Females Four Decades Later. Psychological Science.

[B98-jintelligence-13-00032] Ludwig Monika (1996). Armutskarrieren. Zwischen Abstieg und Aufstieg im Sozialstaat [Poverty Careers. Between Descent and Ascent in the Welfare State].

[B99-jintelligence-13-00032] Maaz Kai, Neumann Marko, Baumert Jürgen (2014). Herkunft und Bildungserfolg von der frühen Kindheit bis ins Erwachsenenalter: Forschungsstand und Interventionsmöglichkeiten aus interdisziplinärer Perspektive [Origin and Educational Success from Early Childhood to Adulthood: State of Research and Possibilities for Intervention from an Interdisciplinary Perspective].

[B100-jintelligence-13-00032] Marks Gary N. (2015). Education, Social Background and Cognitive Ability: The Decline of the Social.

[B101-jintelligence-13-00032] Mayer Karl Ulrich (2009). New Directions in Life Course Research. Annual Review of Sociology.

[B102-jintelligence-13-00032] McElvany Nele, Lorenz Ramona, Frey Andreas, Goldhammer Frank, Schilcher Anita, Stubbe Tobias C. (2023). IGLU 2021: Lesekompetenz von Grundschulkindern im internationalen Vergleich und im Trend über 20 Jahre [Reading Literacy of Elementary School Children in International Comparison and Trend over 20 Years].

[B103-jintelligence-13-00032] Mincer Jacob (1958). Investment in Human Capital and Personal Income Distribution. Journal of Political Economy.

[B104-jintelligence-13-00032] Morgan Stephen L., Kim Young-Mi, Morgan Stephen L., Grusky David B., Fields Gary S. (2006). Chapter Seven. Inequality of Conditions and Intergenerational Mobility: Changing Patterns of Educational Attainment in the United States. Mobility and Inequality.

[B105-jintelligence-13-00032] Mortimer Jeylan T., Staff Jeremy, Oesterle Sabrina, Mortimer Jeylan T., Shanahan Michael J. (2003). Adolescent Work and the Early Socioeconomic Career. Handbook of the Life Course.

[B106-jintelligence-13-00032] Murtza Muhammad Hamid, Gill Shahzad Ali, Aslam Hassan Danial, Noor Amna (2021). Intelligence quotient, job satisfaction, and job performance: The moderating role of personality type. Journal of Public Affairs.

[B107-jintelligence-13-00032] Muthén Linda K., Muthén Bengt O. (2017). Mplus User’s Guide.

[B108-jintelligence-13-00032] Ng Thomas W. H., Eby Lillian T., Sorensen Kelly L., Feldmann Daniel C. (2005). Predictors of objective and subjective career success: A meta-analysis. Personnel Psychology.

[B109-jintelligence-13-00032] OECD (2013). Trends Shaping Education 2013.

[B110-jintelligence-13-00032] OECD (2016a). Excellence and Equity in Education.

[B111-jintelligence-13-00032] OECD (2016b). Trends Shaping Education 2016. Trends Shaping Education.

[B112-jintelligence-13-00032] OECD (2019). Trends Shaping Education 2019. Trends Shaping Education.

[B113-jintelligence-13-00032] OECD (2024). Nurturing Social and Emotional Learning Across the Globe: Findings from the OECD Survey on Social and Emotional Skills 2023.

[B114-jintelligence-13-00032] Pfeffer Fabian T., Smeeding Timothy M., Erkson Robert, Jäntti Markus (2011). Status Attainment and Wealth in the United States and Germany. Persistence, Privilege, and Parenting. The Comparative Study of Intergenerational Mobility.

[B115-jintelligence-13-00032] Rammstedt Beatrice, Helmschrott Susanne, Martin Silke, Massing Natascha, Zabal Anouk (2013). Grundlegende Kompetenzen Erwachsener im internationalen Vergleich: Ergebnisse von PIAAC 2012 [Basic Skills of Adults in International Comparison: Results of the PIAAC 2012].

[B116-jintelligence-13-00032] Rothwell William J., Lindholm John E. (1999). Competency identification, modelling and assessment in the USA. International Journal of Training and Development.

[B117-jintelligence-13-00032] Rothwell William J., Mozaffari Fatemeh, Hajri Azza Al (2025). A Bibliometric Overview of Competency and Capability Modeling: Research Contributions and Trends. Performance Improvement Quarterly.

[B118-jintelligence-13-00032] Rumberger Russell W. (2010). Education and the reproduction of economic inequality in the United States: An empirical investigation. Economics of Education Review.

[B119-jintelligence-13-00032] Schiener Jürgen, Felden Heide von, Schiener Jürgen (2010). Arbeitsmarkt und Berufseinstieg von Akademiker/innen: Theoretische und empirische Grundlagend [Labor market and career entry of academics: Theoretical and empirical foundations]. Transitionen—Übergänge vom Studium in den Beruf.

[B120-jintelligence-13-00032] Schmidt Frank L., Hunter John (2004). General Mental Ability in the World of Work: Occupational Attainment and Job Performance. Journal of Personality and Social Psychology.

[B121-jintelligence-13-00032] Schneider Michael, Preckel Franzis (2017). Variables Associated with Achievement in Higher Education: A Systematic Review of Meta-Analyses. Psychological Bulletin.

[B122-jintelligence-13-00032] Schoon Ingrid (2008). A Transgenerational Model of Status Attainment: The Potential Mediating Role of School Motivation and Education. National Institute Economic Review.

[B123-jintelligence-13-00032] Schoon Ingrid (2010). Childhood cognitive ability and adult academic attainment: Evidence from three British cohort studies. Longitudinal and Life Course Studies.

[B124-jintelligence-13-00032] Schulz Wiebke, Schunck Reinhard, Diewald Martin, Johnson Wendy (2017). Pathways of Intergenerational Transmission of Advantages During Adolescence: Social Background, Cognitive Ability, and Educational Attainment. Journal of Youth and Adolescence.

[B125-jintelligence-13-00032] Settersten Richard A., Gannon Lynn (2005). Structure, Agency, and the Space Between: On the Challenges and Contradictions of a Blended View of the Life Course. Advances in Life Course Research.

[B126-jintelligence-13-00032] Sewell William H., Haller Archibald O., Portes Alejandro (1969). The Educational and Early Occupational Attainment Process. American Sociological Review.

[B127-jintelligence-13-00032] Shambrook Jennifer, Roberts Thomas J., Triscari Robert (2011). Research Administrator Salary: Association with Education, Experience, Credentials and Gender. The Journal of Research Administration.

[B128-jintelligence-13-00032] Shavit Yossi, Müller Walter (2003). From School to Work: A Comparative Study of Educational Qualifications and Occupational Destinations.

[B129-jintelligence-13-00032] Sieppi Antti, Pehkonen Jaakko (2019). Parenthood and gender inequality: Population-based evidence on the child penalty in Finland. Economics Letters.

[B130-jintelligence-13-00032] Solga Heike, Becker Rolf, Solga Heike (2012). Bildung und materielle Ungleichheiten: Der investive Sozialstaat auf dem Prüfstand [Education and Material Inequalities: Putting the Investment Welfare State to the Test].

[B131-jintelligence-13-00032] Sorjonen Kimmo, Hemmingsson Tomas, Lundin Andreas, Falkstedt Daniel, Melin Bo (2012). Intelligence, socioeconomic background, emotional capacity, and level of education as predictors of attained socioeconomic position in a cohort of Swedish men. Intelligence.

[B132-jintelligence-13-00032] Spence Michael (1973). Job Market Signaling. The Quarterly Journal of Economics.

[B133-jintelligence-13-00032] Spengler Marion, Brunner Martin, Damian Rodica I., Lüdtke Oliver, Martin Romain, Roberts Brent W. (2015). Student Characteristics and Behaviors at Age 12 Predict Occupational Success 40 Years Later over and Above Childhood IQ and Parental Socioeconomic Status. Developmental Psychology.

[B134-jintelligence-13-00032] Spengler Marion, Damian Rodica Ioana, Roberts Brent W. (2018). How You Behave in School Predicts Life Success Above and Beyond Family Background, Broad Traits, and Cognitive Ability. Journal of Personality and Social Psychology.

[B135-jintelligence-13-00032] Strenze Tarmo (2007). Intelligence and socioeconomic success: A meta-analytic review of longitudinal research. Intelligence.

[B136-jintelligence-13-00032] Stumm Sophie von, Plomin Robert (2015). Socioeconomic Status and the Growth of Intelligence from Infancy Through Adolescence. Intelligence.

[B137-jintelligence-13-00032] Stumm Sophie von, Macintyre Sally, Batty David G., Clark Heather, Deary Ian J. (2010). Intelligence, social class of origin, childhood behavior disturbance and education as predictors of status attainment in midlife in men: The Aberdeen Children of the 1950s study. Intelligence.

[B138-jintelligence-13-00032] Sweetland Scott R. (1996). Human Capital Theory: Foundations of a Field of Inquiry. Review of Educational Research.

[B139-jintelligence-13-00032] Wai Jonathan (2014). Experts are born, then made: Combining prospective and retrospective longitudinal data shows that cognitive ability matters. Intelligence.

[B140-jintelligence-13-00032] Warren John Robert, Hauser Robert M., Sheridan Jennifer T. (2002). Occupational Stratification across the Life Course: Evidence from the Wisconsin Longitudinal Study. American Sociological Review.

[B141-jintelligence-13-00032] Weber Max (1980). Wirtschaft und Gesellschaft.

[B142-jintelligence-13-00032] Weiss Andrew (1995). Human Capital vs. Signalling Explanations of Wages. Journal of Economic Perspectives.

[B143-jintelligence-13-00032] Wingens Matthias, Wingens Matthias (2022). The Life Course as a Social Construction. Sociological Life Course Research.

[B144-jintelligence-13-00032] Zax Jeffrey S., Rees Daniel I. (2002). IQ, Academic Performance, Environment, and Earnings. Review of Economics and Statistics.

