# Supplementary material for: Occupational Success Across the Lifespan: On the Differential Importance of Childhood Intelligence, Social Background, and Education Across Occupational Development
Source: J Intell. 2025 Mar 6;13(3):32. doi: 10.3390/jintelligence13030032 (PMC11942901; doi:10.3390/jintelligence13030032)
Supplement: Supplementary file 1 [file jintelligence-13-00032-s001.zip › jintelligence-3379552-supplementary.pdf]

Supplemental Material for the article: Occupational Success across the Life Span. On the Differential Importance of Childhood Intelligence, Social Background, and Education across Occupational Development.

By Georg Karl Deutschmann, Michael Becker, and Yi-Jhen Wu

Part 1: M*plus* Syntax for statistical analyses

1: correlations and latent models

```
Title: Correlations
!IQ and SEB latently modelled
!Data and variable Statements are equal across all analyses.

Data:
file = "..\A2_Mplus_v1.5not.dat";
Type = individual;

variable:
names =
case !ID
m_c_sex !Geschlecht
g_stu !Gruppierung: 1=berufl. Abschluss / 2=Studium,uni
t0_G_Bln !T0 Gehalt, ln
t1_G_Bln !T1 Gehalt, ln
t2_G_Bln !T2 Gehalt, ln
t0_BS_IS !T0 Berufsstatus (ISEI)
t1_BS_IS !T1 Berufsstatus (ISEI)
t2_BS_IS !T2 Berufsstatus (ISEI)
p_i_ba !Zertifikat, schulische Bildung
p_i_voc !Zertifikat, berufliche Bildung
p_i_casm !Bildungsjahre (Basis Welle H)
p_i_SNot !Zertifikatsnote, schulische Bildung (gedreht)
p_i_BNo2 !Zertifikatsnote, berufliche Bildung (gedreht)
p_i_BNot !Zertifikatsnote, berufliche Bildung version2 (gedreht)
m_sekh1 !Berufssektor (Welle H) 1=mil+mil
m_sekh2 !Berufssektor (Welle H) 1=roh
m_sekh3 !Berufssektor (Welle H) 1=Bau
m_sekh4 !Berufssektor (Welle H) 1=Nat
m_sekh5 !Berufssektor (Welle H) 1=Log
m_sekh6 !Berufssektor (Welle H) 1=DL
m_sekh7 !Berufssektor (Welle H) 1=Law
m_sekh8 !Berufssektor (Welle H) 1=Heal
m_sekh9 !Berufssektor (Welle H) 1=human
m_seki1 !Berufssektor (Welle i) 1=mil+mil
m_seki2 !Berufssektor (Welle i) 1=roh
m_seki3 !Berufssektor (Welle i) 1=Bau
m_seki4 !Berufssektor (Welle i) 1=Nat
m_seki5 !Berufssektor (Welle i) 1=Log
m_seki6 !Berufssektor (Welle i) 1=DL
m_seki7 !Berufssektor (Welle i) 1=Law
m_seki8 !Berufssektor (Welle i) 1=Heal
m_seki9 !Berufssektor (Welle i) 1=human
m_c_beam !Kontrollvariable, Beamtenstatus, dichotom
m_c_tzh !Teilzeit in Welle H (=1)
m_c_tzi !Teilzeit in Welle I (=1)
m_elt !Im Berufsverlauf elternzeit genannt
m_c_ch !Kinder im Haushalt, dichotom
m_c_migd !Kontrollvariable, Migrationshintergrund, dichotom
m_c_ost !Neue Bundesländer (=1)
p_mu_ASI !SEB: Berufsstatus Mutter, Welle A
p_mu_ESI !SEB: Berufsstatus Mutter, Welle E
p_va_ASI !SEB: Berufsstatus Vater, Welle A
p_va_ESI !SEB: Berufsstatus Vater, Welle E
```

```
p_e_abi !SEB: Anzahl Abiture im Elternhaushalt
p_e_stud !SEB: Anzahl Studienabschlüsse im Elternhaushalt
p_i_ista !IQ: IST55-Skala, Analogien erkennen
p_i_istz !IQ: IST55-Skala, Zahlenreihen
p_i_SkfV !IQ: KFT Sprachanalogien, Welle A
p_i_Wafi !IQ: KFT Figural, Welle A
wl_t1_log !zeit im Beruf T1
wl_t2_log !zeit im Beruf T2
t0_age !Alter zum Berufseinstieg in Jahren
h_wage_n !Stundenlohn Welle H, netto
h_wage_b !Stundenlohn Welle H, brutto
gwt4to7r; !Gewicht

missing = all(-99);
idvariable = case;
weight is gwt4to7r;

usevariables =
t0_G_Bln t1_G_Bln t2_G_Bln t0_BS_IS t1_BS_IS t2_BS_IS p_mu_ASI p_va_ASI p_e_abi
p_e_stud p_i_ista p_i_istz p_i_SkfV p_i_Wafi p_i_casm p_i_SNot p_i_BNot m_c_sex
m_c_ost;

analysis:
type = general;
coverage = .001;
estimator = mlr;

model:
SEB by p_mu_ASI p_va_ASI p_e_Abi p_e_stud;
IQ by p_i_ista p_i_istz p_i_skfv p_i_wafi;

t0_G_Bln t1_G_Bln t2_G_Bln t0_BS_IS t1_BS_IS t2_BS_IS
p_i_casm p_i_Snot p_i_BNot m_c_sex m_c_ost IQ SEB
With
t0_G_Bln t1_G_Bln t2_G_Bln t0_BS_IS t1_BS_IS t2_BS_IS
p_i_casm p_i_Snot p_i_BNot m_c_sex m_c_ost IQ SEB
;
```

2: Regression models without Education as Mediator

```
Title: Occupational Status: Regression without Mediaton

!Data and variable Statements (excluding usevariables) are equal across all
analyses.
!For details see syntax "1:correlations and latent models" in the supplemental
materials

(…)

usevariables =
t0_BS_IS t1_BS_IS t2_BS_IS p_mu_ASI p_va_ASI p_e_abi p_e_stud p_i_ista p_i_istz
p_i_SkfV p_i_Wafi m_c_ost;

analysis:
type = general;
coverage = .001;
estimator = mlr;

model:
SEB by p_mu_ASI p_va_ASI p_e_Abi p_e_stud;
IQ by p_i_ista p_i_istz p_i_skfv p_i_wafi;
t0_BS_IS on SEB IQ m_c_ost;
t1_BS_IS on SEB IQ m_c_ost;
t2_BS_IS on SEB IQ m_c_ost;
```

```
Title: Income: Regression without Mediation

!Data and variable Statements are equal across all analyses.
!For details see syntax “1:correlations and latent models” in the supplemental
materials

(…)

usevariables =
t0_G_Bln t1_G_Bln t2_G_Bln p_mu_ASI p_va_ASI p_e_abi p_e_stud p_i_ista p_i_istz
p_i_SkfV p_i_Wafi m_c_ost;

analysis:
type = general;
coverage = .001;
estimator = mlr;

model:
SEB by p_mu_ASI p_va_ASI p_e_Abi p_e_stud;
IQ by p_i_ista p_i_istz p_i_skfv p_i_wafi;
t0_G_bln on SEB IQ m_c_ost;
t1_G_bln on SEB IQ m_c_ost;
t2_G_bln on SEB IQ m_c_ost;
```

3: Regression Models with Education as Mediator

```
Title: Occupational Status: Regression with Education as Mediator

!Data and variable Statements are equal across all analyses.
!For details see syntax “1:correlations and latent models” in the supplemental
materials

(…)

usevariables =
t0_BS_IS t1_BS_IS t2_BS_IS p_mu_ASI p_va_ASI p_e_abi p_e_stud p_i_ista p_i_istz
p_i_SkfV p_i_Wafi p_i_casm p_i_SNot p_i_BNot m_c_ost;

analysis:
type = general;
coverage = .001;
estimator = mlr;

model:
SEB by p_mu_ASI p_va_ASI p_e_Abi p_e_stud;
IQ by p_i_ista p_i_istz p_i_skfv p_i_wafi;
p_i_casm on SEB IQ m_c_ost;
p_i_SNot on SEB IQ m_c_ost;
p_i_BNot on SEB IQ m_c_ost;
t0_BS_IS on SEB IQ p_i_casm p_i_SNot p_i_BNot m_c_ost;
t1_BS_IS on SEB IQ p_i_casm p_i_SNot p_i_BNot m_c_ost;
t2_BS_IS on SEB IQ p_i_casm p_i_SNot p_i_BNot m_c_ost;
```

```
Title: Income: Regression with Education as Mediator

!Data and variable statements are equal across all analyses.
!For details see syntax “1:correlations and latent models” in the supplemental
materials

(…)

usevariables =
t0_G_Bln t1_G_Bln t2_G_Bln p_mu_ASI p_va_ASI p_e_abi p_e_stud p_i_ista p_i_istz
p_i_SkfV p_i_Wafi p_i_Wdfi p_i_casm p_i_SNot p_i_BNot m_c_sex m_c_ost;

analysis:
type = general;
coverage = .001;
estimator = mlr;

model:
SEB by p_mu_ASI p_va_ASI p_e_Abi p_e_stud;
IQ by p_i_ista p_i_istz p_i_skfv p_i_wafi;
p_i_casm on SEB IQ m_c_sex m_c_ost;
p_i_SNot on SEB IQ m_c_sex m_c_ost;
p_i_BNot on SEB IQ m_c_sex m_c_ost;
t0_G_bln on SEB IQ p_i_casm p_i_SNot p_i_BNot m_c_sex m_c_ost;
t1_G_bln on SEB IQ p_i_casm p_i_SNot p_i_BNot m_c_sex m_c_ost;
t2_G_bln on SEB IQ p_i_casm p_i_SNot p_i_BNot m_c_sex m_c_ost;
```

4: Pathmodels

4.1: Paths only

```
Title: Occupational Status: Autoregressive Paths

!Data and variable statements are equal across all analyses.
!For details see syntax “1:correlations and latent models” in the supplemental
materials

(…)

usevariables =
t0_BS_IS t1_BS_IS t2_BS_IS

analysis:
type = general;
coverage = .001;
estimator = mlr;

model:
t2_BS_IS on t1_BS_IS;
t1_BS_IS on t0_BS_IS;
```

```
Title: Income: Autoregressive Paths

!Data and variable statements are equal across all analyses.
!For details see syntax “1:correlations and latent models” in the supplemental
materials

(…)

usevariables =
t0_G_Bln t1_G_Bln t2_G_Bln

analysis:
type = general;
coverage = .001;
estimator = mlr;

model:
t2_G_bln on t1_G_bln;
t1_G_bln on t0_G_bln;
```

4.2: Regression of Income and Occupational status on IQ, SEB, and autoregressive paths

```
Title: Occupational status: Autoregressive Paths and Predictors

!Data and variable statements are equal across all analyses.
!For details see syntax “1:correlations and latent models” in the supplemental
materials

(…)

usevariables =
t0_BS_IS t1_BS_IS t2_BS_IS p_mu_ASI p_va_ASI p_e_abi p_e_stud p_i_ista p_i_istz
p_i_SkfV p_i_Wafi m_c_ost;

analysis:
type = general;
coverage = .001;
estimator = mlr;

model:
SEB by p_mu_ASI p_va_ASI p_e_Abi p_e_stud;
IQ by p_i_ista p_i_istz p_i_skfv p_i_wafi;
t2_BS_IS on t1_BS_IS;
t1_BS_IS on t0_BS_IS;
t0_BS_IS on SEB IQ m_c_ost;
t1_BS_IS on SEB IQ m_c_ost;
t2_BS_IS on SEB IQ m_c_ost;
```

```
Title: Income: Autoregressive Paths and Predictors

!Data and variable statements are equal across all analyses.
!For details see syntax “1:correlations and latent models” in the supplemental
materials

(…)

usevariables =
t0_G_Bln t1_G_Bln t2_G_Bln p_mu_ASI p_va_ASI p_e_abi p_e_stud p_i_ista p_i_istz
p_i_SkfV p_i_Wafi m_c_ost;

analysis:
type = general;
coverage = .001;
estimator = mlr;

model:
SEB by p_mu_ASI p_va_ASI p_e_Abi p_e_stud;
IQ by p_i_ista p_i_istz p_i_skfv p_i_wafi;
t1_G_bln on t0_G_bln;
t2_G_bln on t1_G_bln;
t0_G_bln on SEB IQ m_c_ost;
t1_G_bln on SEB IQ m_c_ost;
t2_G_bln on SEB IQ m_c_ost;
```

4.3: Mediaton and autoregressive paths

```
Title: Occupational Status: Autoregressive Paths and Education as Mediator

!Data and variable statements are equal across all analyses.
!For details see syntax "1:correlations and latent models" in the supplemental
materials

(…)
usevariables =
t0_BS_IS t1_BS_IS t2_BS_IS p_mu_ASI p_va_ASI p_e_abi p_e_stud p_i_ista p_i_istz
p_i_SkfV p_i_Wafi p_i_casm p_i_SNot p_i_BNot m_c_sex m_c_ost;

analysis:
type = general;
coverage = .001;
estimator = mlr;

model:
SEB by p_mu_ASI p_va_ASI p_e_Abi p_e_stud;
IQ by p_i_ista p_i_istz p_i_skfv p_i_wafi;
t2_BS_IS on t1_BS_IS;
t1_BS_IS on t0_BS_IS;
p_i_casm on SEB IQ m_c_sex m_c_ost;
p_i_SNot on SEB IQ m_c_sex m_c_ost;
p_i_BNot on SEB IQ m_c_sex m_c_ost;
t0_BS_IS on SEB IQ p_i_casm p_i_SNot p_i_BNot m_c_sex m_c_ost;
t1_BS_IS on SEB IQ p_i_casm p_i_SNot p_i_BNot m_c_sex m_c_ost;
t2_BS_IS on SEB IQ p_i_casm p_i_SNot p_i_BNot m_c_sex m_c_ost;
```

```
Title: Income: Autoregressive Paths and Education as Mediator

!Data and variable statements are equal across all analyses.
!For details see syntax "1:correlations and latent models" in the supplemental
materials

(…)
usevariables =
t0_G_Bln t1_G_Bln t2_G_Bln p_mu_ASI p_va_ASI p_e_abi p_e_stud p_i_ista p_i_istz
p_i_SkfV p_i_Wafi p_i_casm p_i_SNot p_i_BNot m_c_sex m_c_ost;

analysis:
type = general;
coverage = .001;
estimator = mlr;

model:
SEB by p_mu_ASI p_va_ASI p_e_Abi p_e_stud;
IQ by p_i_ista p_i_istz p_i_skfv p_i_wafi;
t1_G_bln on t0_G_bln;
t2_G_bln on t1_G_bln;
p_i_casm on SEB IQ m_c_sex m_c_ost;
p_i_SNot on SEB IQ m_c_sex m_c_ost;
p_i_BNot on SEB IQ m_c_sex m_c_ost;
t0_G_bln on SEB IQ p_i_casm p_i_SNot p_i_BNot m_c_sex m_c_ost;
t1_G_bln on SEB IQ p_i_casm p_i_SNot p_i_BNot m_c_sex m_c_ost;
t2_G_bln on SEB IQ p_i_casm p_i_SNot p_i_BNot m_c_sex m_c_ost;
```

5. Testing on change (mediation models)

5.1: Occupational Status

```
Title: Occupational status: Reference model, Education as Mediator

!Data and variable statements are equal across all analyses.
!For details see syntax “1:correlations and latent models” in the supplemental
materials

(…)

usevariables =
t0_BS_IS t1_BS_IS t2_BS_IS p_mu_ASI p_va_ASI p_e_abi p_e_stud p_i_ista p_i_istz
p_i_SkfV p_i_Wafi p_i_casm p_i_SNot p_i_BNot m_c_ost;

analysis:
type = general;
coverage = .001;
estimator = mlr;

model:
SEB by p_mu_ASI p_va_ASI p_e_Abi p_e_stud;
IQ by p_i_ista p_i_istz p_i_skfv p_i_wafi;
p_i_casm on SEB IQ m_c_ost;
p_i_SNot on SEB IQ m_c_ost;
p_i_BNot on SEB IQ m_c_ost;
t0_BS_IS on SEB
            IQ
            p_i_casm
            p_i_SNot p_i_BNot
            m_c_ost;
t1_BS_IS on SEB
            IQ
            p_i_casm
            p_i_SNot p_i_BNot
            m_c_ost;
t2_BS_IS on SEB
            IQ
            p_i_casm
            p_i_SNot p_i_BNot
            m_c_ost;
```

```
Title: Occupational status: SEB Constrained to be equal

!Data variable and analysis statements are equal across all model in the section on
testing on change in moderation models.
!For details see syntax "5.1: Testing on change (moderation models)" in the
supplemental materials

(…)
model:
SEB by p_mu_ASI p_va_ASI p_e_Abi p_e_stud;
IQ by p_i_ista p_i_istz p_i_skfv p_i_wafi;
p_i_casm on SEB IQ m_c_ost;
p_i_SNot on SEB IQ m_c_ost;
p_i_BNot on SEB IQ m_c_ost;
t0_BS_IS on SEB (e1)
            IQ
            p_i_casm
            p_i_SNot p_i_BNot
            m_c_ost;
t1_BS_IS on SEB (e1)
            IQ
            p_i_casm
            p_i_SNot p_i_BNot
            m_c_ost;
t2_BS_IS on SEB (e1)
            IQ
            p_i_casm
            p_i_SNot p_i_BNot
            m_c_ost;



Title: Occupational status: IQ Constrained to be equal

!Data variable and analysis statements are equal across all model in the section on
testing on change in moderation models.
!For details see syntax "5.1: Testing on change (moderation models)" in the
supplemental materials

(…)
model:
SEB by p_mu_ASI p_va_ASI p_e_Abi p_e_stud;
IQ by p_i_ista p_i_istz p_i_skfv p_i_wafi;
p_i_casm on SEB IQ m_c_ost;
p_i_SNot on SEB IQ m_c_ost;
p_i_BNot on SEB IQ m_c_ost;
t0_BS_IS on SEB
            IQ (e1)
            p_i_casm
            p_i_SNot p_i_BNot
            m_c_ost;
t1_BS_IS on SEB
            IQ (e1)
            p_i_casm
            p_i_SNot p_i_BNot
            m_c_ost;
t2_BS_IS on SEB
            IQ (e1)
            p_i_casm
            p_i_SNot p_i_BNot
            m_c_ost;

Title: Occupational status: Education (Casmin) Constrained to be equal
```

```
!Data variable and analysis statements are equal across all model in the section on
testing on change in moderation models.
!For details see syntax "5.1: Testing on change (moderation models)" in the
supplemental materials

(…)
model:
SEB by p_mu_ASI p_va_ASI p_e_Abi p_e_stud;
IQ by p_i_ista p_i_istz p_i_skfv p_i_wafi;
p_i_casm on SEB IQ m_c_ost;
p_i_SNot on SEB IQ m_c_ost;
p_i_BNot on SEB IQ m_c_ost;
t0_BS_IS on SEB
            IQ
            p_i_casm (e1)
            p_i_SNot p_i_BNot
            m_c_ost;
t1_BS_IS on SEB
            IQ
            p_i_casm (e1)
            p_i_SNot p_i_BNot
            m_c_ost;
t2_BS_IS on SEB
            IQ
            p_i_casm (e1)
            p_i_SNot p_i_BNot
            m_c_ost;



Title: Occupational status: GPA gen edu Constrained to be equal

!Data variable and analysis statements are equal across all model in the section on
testing on change in moderation models.
!For details see syntax "5.1: Testing on change (moderation models)" in the
supplemental materials

(…)
model:
SEB by p_mu_ASI p_va_ASI p_e_Abi p_e_stud;
IQ by p_i_ista p_i_istz p_i_skfv p_i_wafi;
p_i_casm on SEB IQ m_c_ost;
p_i_SNot on SEB IQ m_c_ost;
p_i_BNot on SEB IQ m_c_ost;
t0_BS_IS on SEB
            IQ
            p_i_casm
            p_i_SNot (e1) p_i_BNot
            m_c_ost;
t1_BS_IS on SEB
            IQ
            p_i_casm
            p_i_SNot (e1) p_i_BNot
            m_c_ost;
t2_BS_IS on SEB
            IQ
            p_i_casm
            p_i_SNot (e1) p_i_BNot
            m_c_ost;

Title: Occupational status: GPA voc edu Constrained to be equal
```

```
!Data variable and analysis statements are equal across all model in the section on
testing on change in moderation models.
!For details see syntax “5.1: Testing on change (moderation models)” in the
supplemental materials

(…)
model:
SEB by p_mu_ASI p_va_ASI p_e_Abi p_e_stud;
IQ by p_i_ista p_i_istz p_i_skfv p_i_wafi;
p_i_casm on SEB IQ m_c_ost;
p_i_SNot on SEB IQ m_c_ost;
p_i_BNot on SEB IQ m_c_ost;
t0_BS_IS on SEB
            IQ
            p_i_casm
            p_i_SNot p_i_BNot (e1)
            m_c_ost;
t1_BS_IS on SEB
            IQ
            p_i_casm
            p_i_SNot p_i_BNot (e1)
            m_c_ost;
t2_BS_IS on SEB
            IQ
            p_i_casm
            p_i_SNot p_i_BNot (e1)
            m_c_ost;
```

5.2: Income

```
Title: Income: Reference model, Education as Mediator

!Data and variable statements are equal across all analyses.
!For details see syntax “1:correlations and latent models” in the supplemental
materials

(…)

usevariables =
t0_G_bln t1_G_bln t2_G_bln p_mu_ASI p_va_ASI p_e_abi p_e_stud p_i_ista p_i_istz
p_i_SkfV p_i_Wafi p_i_casm p_i_SNot p_i_BNot m_c_ost;

analysis:
type = general;
coverage = .001;
estimator = mlr;

model:
SEB by p_mu_ASI p_va_ASI p_e_Abi p_e_stud;
IQ by p_i_ista p_i_istz p_i_skfv p_i_wafi;
p_i_casm on SEB IQ m_c_ost;
p_i_SNot on SEB IQ m_c_ost;
p_i_BNot on SEB IQ m_c_ost;
t0_G_bln on SEB
            IQ
            p_i_casm
            p_i_SNot p_i_BNot
            m_c_ost;
t1_G_bln on SEB
            IQ
            p_i_casm
            p_i_SNot p_i_BNot
            m_c_ost;
t2_G_bln on SEB
            IQ
            p_i_casm
            p_i_SNot p_i_BNot
            m_c_ost;
```

```
Title: Income: SEB Constrained to be equal

!Data variable and analysis statements are equal across all model in the section on
testing on change in moderation models.
!For details see syntax "5.2: Testing on change (moderation models), income" in the
supplemental materials

(…)

model:
SEB by p_mu_ASI p_va_ASI p_e_Abi p_e_stud;
IQ by p_i_ista p_i_istz p_i_skfv p_i_wafi;
p_i_casm on SEB IQ m_c_ost;
p_i_SNot on SEB IQ m_c_ost;
p_i_BNot on SEB IQ m_c_ost;
t0_G_bln on SEB (e1)
            IQ
            p_i_casm
            p_i_SNot p_i_BNot
            m_c_ost;
t1_G_bln on SEB (e1)
            IQ
            p_i_casm
            p_i_SNot p_i_BNot
            m_c_ost;
t2_G_bln on SEB (e1)
            IQ
            p_i_casm
            p_i_SNot p_i_BNot
            m_c_ost;


Title: Income: IQ Constrained to be equal

!Data variable and analysis statements are equal across all model in the section on
testing on change in moderation models.
!For details see syntax "5.2: Testing on change (moderation models), income" in the
supplemental materials

(…)

model:
SEB by p_mu_ASI p_va_ASI p_e_Abi p_e_stud;
IQ by p_i_ista p_i_istz p_i_skfv p_i_wafi;
p_i_casm on SEB IQ m_c_ost;
p_i_SNot on SEB IQ m_c_ost;
p_i_BNot on SEB IQ m_c_ost;
t0_G_bln on SEB
            IQ (e1)
            p_i_casm
            p_i_SNot p_i_BNot
            m_c_ost;
t1_G_bln on SEB
            IQ (e1)
            p_i_casm
            p_i_SNot p_i_BNot
            m_c_ost;
t2_G_bln on SEB
            IQ (e1)
            p_i_casm
            p_i_SNot p_i_BNot
            m_c_ost;
```

```
Title: Income: Education (Casmin) Constrained to be equal

!Data variable and analysis statements are equal across all model in the section on
testing on change in moderation models.
!For details see syntax "5.2: Testing on change (moderation models), income" in the
supplemental materials

(…)

model:
SEB by p_mu_ASI p_va_ASI p_e_Abi p_e_stud;
IQ by p_i_ista p_i_istz p_i_skfv p_i_wafi;
p_i_casm on SEB IQ m_c_ost;
p_i_SNot on SEB IQ m_c_ost;
p_i_BNot on SEB IQ m_c_ost;
t0_G_bln on SEB
            IQ
            p_i_casm (e1)
            p_i_SNot p_i_BNot
            m_c_ost;
t1_G_bln on SEB
            IQ
            p_i_casm (e1)
            p_i_SNot p_i_BNot
            m_c_ost;
t2_G_bln on SEB
            IQ
            p_i_casm (e1)
            p_i_SNot p_i_BNot
            m_c_ost;


Title: Income: GPA gen edu Constrained to be equal

!Data variable and analysis statements are equal across all model in the section on
testing on change in moderation models.
!For details see syntax "5.2: Testing on change (moderation models), income" in the
supplemental materials

(…)

model:
SEB by p_mu_ASI p_va_ASI p_e_Abi p_e_stud;
IQ by p_i_ista p_i_istz p_i_skfv p_i_wafi;
p_i_casm on SEB IQ m_c_ost;
p_i_SNot on SEB IQ m_c_ost;
p_i_BNot on SEB IQ m_c_ost;
t0_G_bln on SEB
            IQ
            p_i_casm
            p_i_SNot (e1) p_i_BNot
            m_c_ost;
t1_G_bln on SEB
            IQ
            p_i_casm
            p_i_Snot (e1) p_i_BNot
            m_c_ost;
t2_G_bln on SEB
            IQ
            p_i_casm
            p_i_Snot (e1) p_i_BNot
            m_c_ost;
```

```
Title: Income: GPA voc edu Constrained to be equal

!Data variable and analysis statements are equal across all model in the section on
testing on change in moderation models.
!For details see syntax “5.2: Testing on change (moderation models), income” in the
supplemental materials

(…)

model:
SEB by p_mu_ASI p_va_ASI p_e_Abi p_e_stud;
IQ by p_i_ista p_i_istz p_i_skfv p_i_wafi;
p_i_casm on SEB IQ m_c_ost;
p_i_SNot on SEB IQ m_c_ost;
p_i_BNot on SEB IQ m_c_ost;
t0_G_bln on SEB
            IQ
            p_i_casm
            p_i_SNot p_i_BNot (e1)
            m_c_ost;
t1_G_bln on SEB
            IQ
            p_i_casm
            p_i_Snot p_i_BNot (e1)
            m_c_ost;
t2_G_bln on SEB
            IQ
            p_i_casm
            p_i_Snot p_i_BNot (e1)
            m_c_ost;
```

6. Testing on change (path models)

6.1: Occupational Status

```
Title: Occupational status: Reference model, Education as Mediator

!Data and variable statements are equal across all analyses.
!For details see syntax “1:correlations and latent models” in the supplemental
materials

(…)

usevariables =
t0_BS_IS t1_BS_IS t2_BS_IS p_mu_ASI p_va_ASI p_e_abi p_e_stud p_i_ista p_i_istz
p_i_SkfV p_i_Wafi p_i_casm p_i_SNot p_i_BNot m_c_ost;

analysis:
type = general;
coverage = .001;
estimator = mlr;

model:
SEB by p_mu_ASI p_va_ASI p_e_Abi p_e_stud;
IQ by p_i_ista p_i_istz p_i_skfv p_i_wafi;
t2_BS_IS on t1_BS_IS;
t1_BS_IS on t0_BS_IS;
p_i_casm on SEB IQ m_c_ost;
p_i_SNot on SEB IQ m_c_ost;
p_i_BNot on SEB IQ m_c_ost;
t0_BS_IS on SEB
            IQ
```

```
            p_i_casm
            p_i_SNot p_i_BNot
            m_c_ost;
t1_BS_IS on SEB
            IQ
            p_i_casm
            p_i_SNot p_i_BNot
            m_c_ost;
t2_BS_IS on SEB
            IQ
            p_i_casm
            p_i_SNot p_i_BNot
            m_c_ost;
```

```
Title: Occupational status: SEB Constrained to be equal

!Data variable and analysis statements are equal across all model in the section on
testing on change in moderation models.
!For details see syntax "5.1: Testing on change (moderation models)" in the
supplemental materials

(…)
model:
SEB by p_mu_ASI p_va_ASI p_e_Abi p_e_stud;
IQ by p_i_ista p_i_istz p_i_skfv p_i_wafi;
t2_BS_IS on t1_BS_IS;
t1_BS_IS on t0_BS_IS;
p_i_casm on SEB IQ m_c_ost;
p_i_SNot on SEB IQ m_c_ost;
p_i_BNot on SEB IQ m_c_ost;
t0_BS_IS on SEB (e1)
            IQ
            p_i_casm
            p_i_SNot p_i_BNot
            m_c_ost;
t1_BS_IS on SEB (e1)
            IQ
            p_i_casm
            p_i_SNot p_i_BNot
            m_c_ost;
t2_BS_IS on SEB (e1)
            IQ
            p_i_casm
            p_i_SNot p_i_BNot
            m_c_ost;



Title: Occupational status: IQ Constrained to be equal

!Data variable and analysis statements are equal across all model in the section on
testing on change in moderation models.
!For details see syntax "5.1: Testing on change (moderation models)" in the
supplemental materials

(…)
model:
SEB by p_mu_ASI p_va_ASI p_e_Abi p_e_stud;
IQ by p_i_ista p_i_istz p_i_skfv p_i_wafi;
t2_BS_IS on t1_BS_IS;
t1_BS_IS on t0_BS_IS;
p_i_casm on SEB IQ m_c_ost;
p_i_SNot on SEB IQ m_c_ost;
p_i_BNot on SEB IQ m_c_ost;
t0_BS_IS on SEB
            IQ (e1)
            p_i_casm
            p_i_SNot p_i_BNot
            m_c_ost;
t1_BS_IS on SEB
            IQ (e1)
            p_i_casm
            p_i_SNot p_i_BNot
            m_c_ost;
t2_BS_IS on SEB
            IQ (e1)
            p_i_casm
```

```
            p_i_SNot p_i_BNot
            m_c_ost;

Title: Occupational status: Education (Casmin) Constrained to be equal

!Data variable and analysis statements are equal across all model in the section on
testing on change in moderation models.
!For details see syntax “5.1: Testing on change (moderation models)” in the
supplemental materials

(…)
model:
SEB by p_mu_ASI p_va_ASI p_e_Abi p_e_stud;
IQ by p_i_ista p_i_istz p_i_skfv p_i_wafi;
t2_BS_IS on t1_BS_IS;
t1_BS_IS on t0_BS_IS;
p_i_casm on SEB IQ m_c_ost;
p_i_SNot on SEB IQ m_c_ost;
p_i_BNot on SEB IQ m_c_ost;
t0_BS_IS on SEB
            IQ
            p_i_casm (e1)
            p_i_SNot p_i_BNot
            m_c_ost;
t1_BS_IS on SEB
            IQ
            p_i_casm (e1)
            p_i_SNot p_i_BNot
            m_c_ost;
t2_BS_IS on SEB
            IQ
            p_i_casm (e1)
            p_i_SNot p_i_BNot
            m_c_ost;



Title: Occupational status: GPA gen edu Constrained to be equal

!Data variable and analysis statements are equal across all model in the section on
testing on change in moderation models.
!For details see syntax “5.1: Testing on change (moderation models)” in the
supplemental materials

(…)
model:
SEB by p_mu_ASI p_va_ASI p_e_Abi p_e_stud;
IQ by p_i_ista p_i_istz p_i_skfv p_i_wafi;
t2_BS_IS on t1_BS_IS;
t1_BS_IS on t0_BS_IS;
p_i_casm on SEB IQ m_c_ost;
p_i_SNot on SEB IQ m_c_ost;
p_i_BNot on SEB IQ m_c_ost;
t0_BS_IS on SEB
            IQ
            p_i_casm
            p_i_SNot (e1) p_i_BNot
            m_c_ost;
t1_BS_IS on SEB
            IQ
            p_i_casm
            p_i_SNot (e1) p_i_BNot
            m_c_ost;
t2_BS_IS on SEB
```

```
            IQ
            p_i_casm
            p_i_SNot (e1) p_i_BNot
            m_c_ost;

Title: Occupational status: GPA voc edu Constrained to be equal

!Data variable and analysis statements are equal across all model in the section on
testing on change in moderation models.
!For details see syntax "5.1: Testing on change (moderation models)" in the
supplemental materials

(…)
model:
SEB by p_mu_ASI p_va_ASI p_e_Abi p_e_stud;
IQ by p_i_ista p_i_istz p_i_skfv p_i_wafi;
t2_BS_IS on t1_BS_IS;
t1_BS_IS on t0_BS_IS;
p_i_casm on SEB IQ m_c_ost;
p_i_SNot on SEB IQ m_c_ost;
p_i_BNot on SEB IQ m_c_ost;
t0_BS_IS on SEB
            IQ
            p_i_casm
            p_i_SNot p_i_BNot (e1)
            m_c_ost;
t1_BS_IS on SEB
            IQ
            p_i_casm
            p_i_SNot p_i_BNot (e1)
            m_c_ost;
t2_BS_IS on SEB
            IQ
            p_i_casm
            p_i_SNot p_i_BNot (e1)
            m_c_ost;
```

6.2: Income

```
Title: Income: Reference model, Education as Mediator

!Data and variable statements are equal across all analyses.
!For details see syntax "1:correlations and latent models" in the supplemental
materials

(…)

usevariables =
t0_G_bln t1_G_bln t2_G_bln p_mu_ASI p_va_ASI p_e_abi p_e_stud p_i_ista p_i_istz
p_i_SkfV p_i_Wafi p_i_casm p_i_SNot p_i_BNot m_c_ost;

analysis:
type = general;
coverage = .001;
estimator = mlr;

model:
SEB by p_mu_ASI p_va_ASI p_e_Abi p_e_stud;
IQ by p_i_ista p_i_istz p_i_skfv p_i_wafi;
t2_BS_IS on t1_BS_IS;
t1_BS_IS on t0_BS_IS;
p_i_casm on SEB IQ m_c_ost;
p_i_SNot on SEB IQ m_c_ost;
p_i_BNot on SEB IQ m_c_ost;
t0_G_bln on SEB
            IQ
            p_i_casm
            p_i_SNot p_i_BNot
            m_c_ost;
t1_G_bln on SEB
            IQ
            p_i_casm
            p_i_SNot p_i_BNot
            m_c_ost;
t2_G_bln on SEB
            IQ
            p_i_casm
            p_i_SNot p_i_BNot
            m_c_ost;
```

```
Title: Income: SEB Constrained to be equal

!Data variable and analysis statements are equal across all model in the section on
testing on change in moderation models.
!For details see syntax “5.2: Testing on change (moderation models), income” in the
supplemental materials

(…)

model:
SEB by p_mu_ASI p_va_ASI p_e_Abi p_e_stud;
IQ by p_i_ista p_i_istz p_i_skfv p_i_wafi;
t2_BS_IS on t1_BS_IS;
t1_BS_IS on t0_BS_IS;
p_i_casm on SEB IQ m_c_ost;
p_i_SNot on SEB IQ m_c_ost;
p_i_BNot on SEB IQ m_c_ost;
t0_G_bln on SEB (e1)
            IQ
            p_i_casm
            p_i_SNot p_i_BNot
            m_c_ost;
t1_G_bln on SEB (e1)
            IQ
            p_i_casm
            p_i_SNot p_i_BNot
            m_c_ost;
t2_G_bln on SEB (e1)
            IQ
            p_i_casm
            p_i_SNot p_i_BNot
            m_c_ost;


Title: Income: IQ Constrained to be equal

!Data variable and analysis statements are equal across all model in the section on
testing on change in moderation models.
!For details see syntax “5.2: Testing on change (moderation models), income” in the
supplemental materials

(…)

model:
SEB by p_mu_ASI p_va_ASI p_e_Abi p_e_stud;
IQ by p_i_ista p_i_istz p_i_skfv p_i_wafi;
t2_BS_IS on t1_BS_IS;
t1_BS_IS on t0_BS_IS;
p_i_casm on SEB IQ m_c_ost;
p_i_SNot on SEB IQ m_c_ost;
p_i_BNot on SEB IQ m_c_ost;
t0_G_bln on SEB
            IQ (e1)
            p_i_casm
            p_i_SNot p_i_BNot
            m_c_ost;
t1_G_bln on SEB
            IQ (e1)
            p_i_casm
            p_i_SNot p_i_BNot
            m_c_ost;
t2_G_bln on SEB
            IQ (e1)
            p_i_casm
```

```
            p_i_SNot p_i_BNot
            m_c_ost;


Title: Income: Education (Casmin) Constrained to be equal

!Data variable and analysis statements are equal across all model in the section on
testing on change in moderation models.
!For details see syntax "5.2: Testing on change (moderation models), income" in the
supplemental materials

(…)

model:
SEB by p_mu_ASI p_va_ASI p_e_Abi p_e_stud;
IQ by p_i_ista p_i_istz p_i_skfv p_i_wafi;
t2_BS_IS on t1_BS_IS;
t1_BS_IS on t0_BS_IS;
p_i_casm on SEB IQ m_c_ost;
p_i_SNot on SEB IQ m_c_ost;
p_i_BNot on SEB IQ m_c_ost;
t0_G_bln on SEB
            IQ
            p_i_casm (e1)
            p_i_SNot p_i_BNot
            m_c_ost;
t1_G_bln on SEB
            IQ
            p_i_casm (e1)
            p_i_SNot p_i_BNot
            m_c_ost;
t2_G_bln on SEB
            IQ
            p_i_casm (e1)
            p_i_SNot p_i_BNot
            m_c_ost;


Title: Income: GPA gen edu Constrained to be equal

!Data variable and analysis statements are equal across all model in the section on
testing on change in moderation models.
!For details see syntax "5.2: Testing on change (moderation models), income" in the
supplemental materials

(…)

model:
SEB by p_mu_ASI p_va_ASI p_e_Abi p_e_stud;
IQ by p_i_ista p_i_istz p_i_skfv p_i_wafi;
t2_BS_IS on t1_BS_IS;
t1_BS_IS on t0_BS_IS;
p_i_casm on SEB IQ m_c_ost;
p_i_SNot on SEB IQ m_c_ost;
p_i_BNot on SEB IQ m_c_ost;
t0_G_bln on SEB
            IQ
            p_i_casm
            p_i_SNot (e1) p_i_BNot
            m_c_ost;
t1_G_bln on SEB
            IQ
            p_i_casm
            p_i_Snot (e1) p_i_BNot
```

```
            m_c_ost;
t2_G_bln on SEB
            IQ
            p_i_casm
            p_i_Snot (e1) p_i_BNot
            m_c_ost;


Title: Income: GPA voc edu Constrained to be equal

!Data variable and analysis statements are equal across all model in the section on
testing on change in moderation models.
!For details see syntax "5.2: Testing on change (moderation models), income" in the
supplemental materials

(…)

model:
SEB by p_mu_ASI p_va_ASI p_e_Abi p_e_stud;
IQ by p_i_ista p_i_istz p_i_skfv p_i_wafi;
t2_BS_IS on t1_BS_IS;
t1_BS_IS on t0_BS_IS;
p_i_casm on SEB IQ m_c_ost;
p_i_SNot on SEB IQ m_c_ost;
p_i_BNot on SEB IQ m_c_ost;
t0_G_bln on SEB
            IQ
            p_i_casm
            p_i_SNot p_i_BNot (e1)
            m_c_ost;
t1_G_bln on SEB
            IQ
            p_i_casm
            p_i_Snot p_i_BNot (e1)
            m_c_ost;
t2_G_bln on SEB
            IQ
            p_i_casm
            p_i_Snot p_i_BNot (e1)
            m_c_ost;
```
